# The PI3K/AKT/mTOR pathway in scar remodeling and keloid formation: mechanisms and therapeutic perspectives

**DOI:** 10.3389/fphar.2025.1678953

**Published:** 2025-12-04

**Authors:** Jing Liu, Junfei Yan, Shengbin Qi, Jie Zhang, Xin Zhang, Yaping Zhao

**Affiliations:** Plastic Surgery Center, Shijiazhuang People’s Hospital, Shijiazhuang, China

**Keywords:** keloid, hypertrophic scar, PI3K/Akt/mTOR pathway, fibroblast proliferation, extracellular matrix, non-coding RNAs, targeted therapy, drug delivery platforms

## Abstract

Keloids and hypertrophic scars (HTS) represent aberrant wound healing characterized by excessive fibroblast activity and extracellular matrix accumulation. The PI3K/AKT/mTOR signaling pathway is vital in regulating these processes, promoting fibroblast proliferation, survival, and collagen synthesis. Dysregulation of this pathway, driven by genetic mutations, post-transcriptional modulation, and upstream signaling, contributes significantly to the pathogenesis of pathological scarring. This review collects current knowledge on the molecular mechanisms underlying PI3K/AKT/mTOR activation in keloids and HTS, highlighting the roles of key regulators such as PTEN, NEDD4, and non-coding RNAs. It also evaluates therapeutic strategies targeting this axis, including small-molecule inhibitors, natural compounds, and emerging delivery platforms. Targeting PI3K/AKT/mTOR offers a compelling avenue for developing effective, mechanism-based keloid and hypertrophic scarring treatments. The PI3K/AKT/mTOR signaling axis is central to these cellular mechanisms, which drive fibroblast proliferation, survival, myofibroblast transdifferentiation, and metabolic reprogramming (including suppressed autophagy and enhanced glycolysis.

## Graphical Abstract

**Figure:**
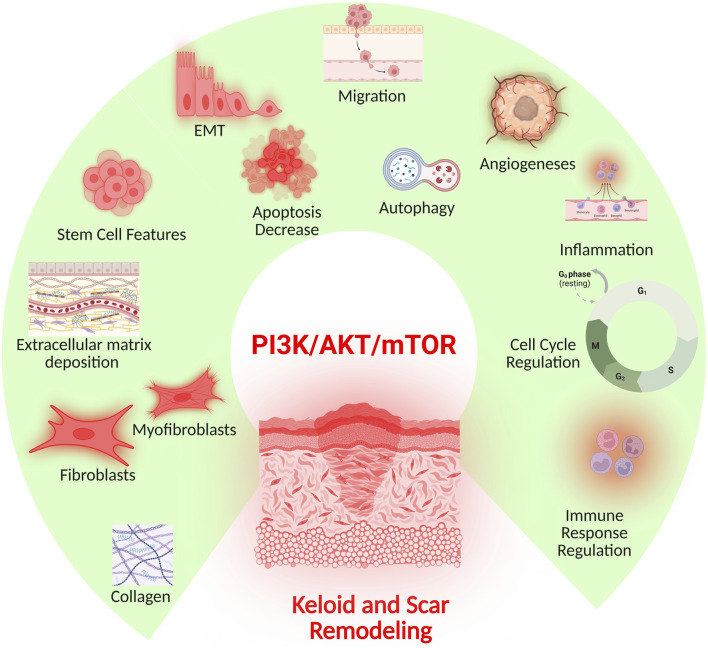

## 1 Introduction

Keloids are abnormal overgrowths of scar tissue that form at the site of a skin injury, often extending beyond the original wound boundaries. Unlike normal scars, which typically fade over time, keloids continue to grow and can become large, raised, and thickened (Limandjaja et al., 2021). They are usually pink, red, or darker than the surrounding skin and may cause itching, pain, and tenderness (Bran et al., 2009). Keloids are most common in individuals with darker skin tones and can occur after even minor skin trauma, such as cuts, burns, or piercings (Shaheen, 2017). Due to their persistent and aggressive growth, keloids can be disfiguring and may limit movement if they develop in areas of high tension, significantly impacting a person’s quality of life (Fang et al., 2025). The formal therapies for keloid treatment aim to reduce scar formation, alleviate symptoms, and improve cosmetic appearance. These include corticosteroid injections, which are commonly used to reduce inflammation and collagen production in the affected area, helping to flatten the keloid (Kim, 2021). Surgical excision is another option, where the keloid is surgically removed, although it may sometimes lead to recurrence (Gold et al., 2020). Pressure therapy, using specially designed garments to apply constant pressure on the scar, can also effectively prevent keloid growth post-surgery (Ojeh et al., 2020). Additionally, laser therapy, including fractional laser treatments, is used to improve the texture and color of the keloid (Mamalis et al., 2014). Cryotherapy and radiotherapy may also be utilized in some cases, particularly for recurring keloids (Ekstein et al., 2021). These therapies can be effective but often require multiple treatments and may only provide partial success. Combining these therapies may sometimes be necessary to achieve the best results (Tai et al., 2023).

The PI3K/AKT/mTOR signaling cascade plays a vital role in controlling key cellular functions such as cell growth, survival, metabolism, and proliferation. This pathway is initiated by the activation of phosphoinositide 3-kinase (PI3K), which facilitates the transformation of phosphatidylinositol (Fang et al., 2025; Kim, 2021)-bisphosphate (PIP2) into phosphatidylinositol (Shaheen, 2017; Fang et al., 2025; Kim, 2021)-trisphosphate (PIP3) at the plasma membrane (Yu and Cui, 2016). This process triggers the recruitment and activation of AKT, a serine/threonine kinase also referred to as protein kinase B, which subsequently modulates multiple downstream effectors that govern cell survival, metabolic activity, and protein production (Song et al., 2005). A primary downstream target of AKT is the mechanistic target of rapamycin (mTOR), a core controller of cellular growth and metabolic function (Gibbons et al., 2009). mTOR operates within two distinct complexes, mTORC1 and mTORC2, which oversee critical processes such as protein synthesis and autophagy, ultimately supporting cell growth and viability (Ballesteros‐Álvarez and Andersen, 2021). Abnormal regulation of this signaling pathway has been linked to numerous diseases, such as cancer, diabetes, and fibrosis, due to its central role in driving uncontrolled cell growth and survival under pathological circumstances (Qin et al., 2021). The PI3K/AKT/mTOR signaling pathway is deeply involved in keloid development, as it controls essential mechanisms such as fibroblast growth, collagen production, and fibrotic tissue formation (Roy et al., 2023). Phosphorylation of the PI3K/AKT pathway leads to AKT activation, which in turn stimulates the mTORC1 complex. This activation suppresses autophagy and results in the buildup of damaged mitochondria and increased reactive oxygen species (ROS) (Kma and Baruah, 2022). Disruption of the PI3K/AKT/mTOR pathway fosters persistent inflammation, activates fibroblasts, and drives excessive collagen deposition, hallmarks of keloid formation (Tan et al., 2019). Elevated activity of the PI3K/AKT/mTOR pathway hinders the removal of damaged mitochondria by disrupting mitochondrial autophagy (mitophagy), thereby intensifying fibrosis (Sun et al., 2021). Consequently, inhibiting this signaling cascade presents a promising therapeutic approach for reducing pathological scar formation in keloid disorders (Liu et al., 2024). Therefore, this review aims to comprehensively summarize the molecular mechanisms by which the PI3K/AKT/mTOR signaling pathway contributes to the development and persistence of keloids and hypertrophic scars, and to evaluate current and emerging therapeutic strategies targeting this axis for scar remodeling and treatment.

## 2 Keloid pathogenesis and signaling pathways

Keloid development is a multifaceted process influenced by genetic factors, immune system imbalances, and disrupted wound healing. It typically starts with persistent inflammation and hyperactivation of fibroblasts, resulting in excessive ECM accumulation, especially disorganized type I and III collagen (Potekaev et al., 2021; Jiang et al., 2024). The pathogenesis of keloid formation is complex and multifactorial, involving genetic susceptibility, epigenetic alterations, endocrine influences, and dysregulated wound-healing responses (Latoni et al., 2024). After skin injury, persistent inflammation and an imbalance in cytokine signaling, particularly elevated IL-6, IL-13, and TGF-β, promote fibroblast proliferation, differentiation into myofibroblasts, and excessive extracellular matrix deposition (Johnson et al., 2020). Aberrant activation of pathways such as JAK/STAT, TGF-β/SMAD, HIF-1α, VEGF, and NOTCH further enhances collagen synthesis, angiogenesis, and resistance to apoptosis, leading to the formation of thick, disorganized collagen bundles. In addition, mechanical tension aggravates the condition via integrin and YAP/TAZ pathways, reinforcing a self-sustaining cycle of fibrosis (Latoni et al., 2024). Genetic influences, including cytokine receptor polymorphisms and aberrant regulation of non-coding RNAs, play a role in driving the excessive fibroproliferative response, leading to thick, elevated scars that grow beyond the initial wound margins (Kim and Kim, 2024). Epigenetic regulators, including DNA methylation changes and noncoding RNAs, along with hormonal and renin–angiotensin system effects, amplify these processes. The result is uncontrolled scar growth that extends beyond the wound margin, distinguishing keloids from hypertrophic scars and accounting for their chronicity, invasiveness, and poor response to spontaneous resolution (Latoni et al., 2024; Lv et al., 2020). Immune cell infiltration, particularly M2 macrophages, Th2 cells, and Tregs, sustains a profibrotic environment, while genetic susceptibility, epigenetic modifications, and dysregulated noncoding RNAs further amplify pathogenic signaling. Together, these interactions give keloids cancer-like behaviors, persistent growth, invasiveness, and recurrence, highlighting the importance of targeting cellular crosstalk and microenvironmental regulation in future therapies (Zhang M. et al., 2023). The PI3K/Akt signaling pathway plays a central role in skin homeostasis by regulating keratinocyte proliferation, differentiation, apoptosis, angiogenesis, metabolism, and wound repair. Its normal activation maintains the epidermal barrier, supports hair follicle stem cell renewal, promotes wound healing via EMT, and protects melanocytes from oxidative stress (Teng et al., 2021). The PI3K/AKT/mTOR pathway is a central signaling cascade that integrates growth factor, nutrient, and energy cues to control cell growth, survival, metabolism, and autophagy (Xu Z. et al., 2020). When receptor tyrosine kinases, cytokine receptors, or GPCRs are activated, PI3K generates PIP_3_ at the plasma membrane, which recruits AKT and PDK1; AKT becomes fully active after phosphorylation by PDK1 (Thr308) and mTORC2 (Ser473) (He et al., 2021). Active AKT promotes survival by inhibiting pro-apoptotic proteins (e.g., BAD, caspase-9) and FOXO transcription factors (Pungsrinont et al., 2021), while simultaneously driving growth by inactivating the TSC1/2 complex and PRAS40, which unleashes Rheb to activate mTORC1 (de la Calle Arregui et al., 2021). Once active, mTORC1 stimulates protein and lipid synthesis, enhances glucose metabolism, and suppresses autophagy, thereby ensuring biosynthesis outweighs catabolism when nutrients are abundant (Deleyto-Seldas and Efeyan, 2021). Crosstalk with MAPK, Wnt/β-catenin, Notch, HIF, and Rho GTPase pathways further integrates environmental signals, while negative regulators like PTEN and AMPK prevent unchecked activity (Roy et al., 2023). Dysregulation of this pathway, however, contributes not only to skin malignancies (e.g., melanoma, BCC, SCC) but also to a wide range of non-malignant skin disorders. Specifically, it promotes lipogenesis and inflammation in acne, keratinocyte hyperproliferation and cytokine imbalance in psoriasis, T-cell dysfunction in atopic dermatitis, fibrosis in scleroderma, fibroblast overactivation in keloids, melanocyte apoptosis in vitiligo, and hair follicle stem cell apoptosis in androgenic alopecia (Teng et al., 2021). At the tissue scale, the axis coordinates homeostasis by balancing proliferation with differentiation and death (Teng et al., 2021). In skin, AKT/mTOR activity supports keratinocyte survival and differentiation (Buerger et al., 2017), integrates cytokine and growth-factor signals (IL-1/IL-23/IL-36; EGFR/IGF-1R) (Roy et al., 2023), and couples metabolic state to barrier formation; dysregulation skews toward hyperproliferation, aberrant differentiation, angiogenesis, and inflammation. This same wiring explains why inhibitors at different tiers (PI3K, dual PI3K/mTOR, mTORC1/C2) show disease-modifying activity yet also exhibit context-dependent feedback, necessitating vertical (multi-node) strategies for durable control (Roy et al., 2023).

## 3 Molecular mechanisms of PI3K/AKT/mTOR pathway dysregulation in keloid

**FIGURE 1:**
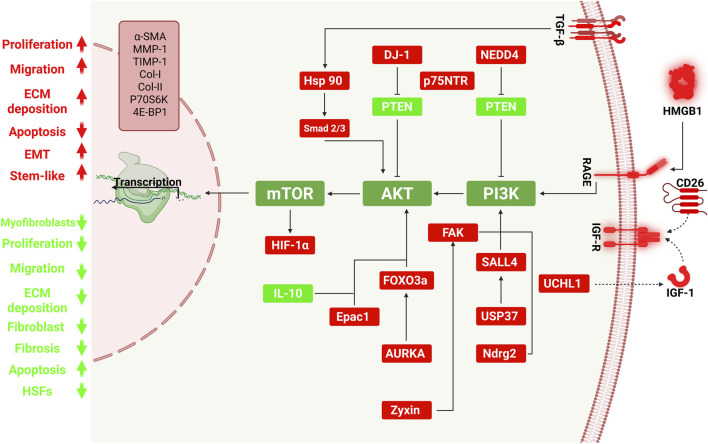
Molecular regulation of the PI3K/AKT/mTOR pathway in keloid fibroblast activation and fibrosis. This schematic illustrates key upstream regulators, intracellular signaling molecules, and downstream transcriptional outcomes involved in the dysregulation of the PI3K/AKT/mTOR pathway during keloid pathogenesis. Activation of receptor complexes such as TGF-βR, IGF-1R, CD26, and RAGE by extracellular ligands (e.g., TGF-β, IGF-1, HMGB1) initiates PI3K/AKT signaling, which promotes fibroblast proliferation, migration, ECM deposition, apoptosis resistance, and myofibroblast differentiation. Positive regulators (shown in red), including Hsp90, DJ-1, NEDD4, UCHL1, CD26, USP37, SALL4, AURKA, Epac1, Zyxin, Ndrg2, p75NTR, and FAK, enhance pathway activation and drive fibrotic responses. In contrast, PTEN and IL-10 (green) serve as negative regulators, limiting PI3K/AKT/mTOR activity and fibrotic remodeling. mTOR, via its complexes mTORC1 and mTORC2, further regulates protein synthesis, cell survival, and HIF-1α-mediated transcription. The cumulative effects of these signaling events include upregulation of profibrotic genes such as α-SMA, TIMP-1, collagen I/III, and P70S6K, promoting the keloid phenotype characterized by excessive and persistent scar formation. Arrows indicate stimulatory effects; blunt arrows denote inhibitory interactions. The left panel summarizes functional outcomes of pathway activation (red) versus inhibition (green).

The PI3K/AKT/mTOR signaling pathway is a crucial molecular network that regulates various cellular processes, including metabolism, growth, survival, and differentiation (Mercurio et al., 2021). Activation of the pathway begins when receptor tyrosine kinases (RTKs) stimulate PI3K, leading to the generation of PIP3, a lipid-based secondary messenger. PIP3 facilitates the membrane recruitment of AKT, which is subsequently phosphorylated and activated by PDK1 and mTORC2 (Roy et al., 2023). Once activated, AKT phosphorylates multiple downstream effectors that enhance cell survival, growth, and metabolic activity (Yu and Cui, 2016). mTOR functions through two distinct complexes: mTORC1, which controls protein synthesis and cellular growth, and mTORC2, which plays a role in promoting cell survival and organizing the cytoskeleton (Szwed et al., 2021). Aberrant activity of this pathway, frequently due to mutations in genes such as PIK3CA, is associated with a range of diseases, including cancers, metabolic syndromes, and neurodegenerative disorders (Omolekan et al., 2024). This section explores the molecular mechanisms by which PI3K/AKT/mTOR pathway dysregulation contributes to hypertrophic scar formation, highlighting its role in persistent fibroblast activation and abnormal tissue remodeling (Figure 1). The following sections examine how various modulators regulate the PI3K/AKT/mTOR pathway during the progression of keloids and hypertrophic scars.

### 3.1 High-mobility group box 1

High-mobility group box 1 (HMGB1) drives keloid pathogenesis by enhancing fibroblast proliferation, collagen synthesis, and myofibroblast differentiation, primarily through activation of RAGE-MAPK, NF-κB, and AKT signaling. This process upregulates profibrotic markers and suppresses ECM-degrading enzymes, thereby promoting fibroblast migration and persistent fibrosis; inhibition with glycyrrhizic acid attenuates these effects (Zhao et al., 2018; Huang and Ogawa, 2022; Lee et al., 2018; Kim et al., 2017; Radziszewski et al., 2024; Baek et al., 2024; Shen et al., 2024).

### 3.2 PTEN

PTEN suppresses hypertrophic scar fibroblast proliferation and collagen synthesis by inhibiting PI3K/AKT signaling, but its downregulation in scars activates AKT-driven fibrogenesis. Overexpression of PTEN selectively inhibits HSFBs without affecting normal fibroblasts, underscoring its regulatory role. In keloids, DJ-1 inactivates PTEN via redox-dependent S-nitrosylation, thereby activating PI3K/AKT/mTOR signaling, which enhances fibroblast proliferation, migration, invasion, and collagen overproduction (Guo et al., 2012; Li et al., 2024; Lv et al., 2023).

### 3.3 NEDD4

NEDD4 promotes keloid progression by ubiquitinating and degrading PTEN, thereby hyperactivating the PI3K/AKT pathway. This leads to downstream β-catenin accumulation, suppression of cell cycle inhibitors, and enhanced fibroblast proliferation, migration, and ECM overproduction. In keloids, PTEN expression is markedly reduced, and its inverse correlation with NEDD4-1 highlights post-translational regulation as a key driver of aberrant fibroblast growth and tissue remodeling (Sang et al., 2015; Wang et al., 2007; Chung et al., 2011; Chen et al., 2018).

### 3.4 IGF-1

UCHL1 aggravates keloid fibrosis by inducing IGF-1 secretion and activating the PI3K/Akt/mTOR/HIF-1α pathway, thereby enhancing collagen I and α-SMA expression independent of fibroblast proliferation or migration; its expression is further upregulated by TGF-β1 from M2 macrophages, creating a pro-fibrotic feedback loop. Similarly, CD26 drives keloid fibroblast proliferation and invasion through IGF-1/IGF-1R-mediated PI3K/Akt/mTOR activation, promoting mTOR downstream effectors (P70S6K, 4E-BP1); inhibition of CD26 or IGF-1R abrogates these effects, underscoring their central role in keloid progression (Guo et al., 2023; Wang and Liu, 2024; Xin et al., 2020).

### 3.5 USP37

USP37 promotes keloid pathogenesis by stabilizing SALL4 through deubiquitination, thereby sustaining PI3K/AKT activation and driving fibroblast growth, migration, invasion, glycolysis, and ECM accumulation. Inhibition of USP37 or PI3K/AKT signaling attenuates these effects, highlighting the USP37/SALL4 axis as a critical regulator of keloid progression (Fang et al., 2025; Xu et al., 2023).

### 3.6 Ndrg2

NDRG2 is upregulated in hypertrophic scar tissue and experimental fibrosis models, where it enhances fibroblast proliferation and migration by activating the PI3K/AKT pathway. Overexpression increases PI3K/AKT phosphorylation, while NDRG2 silencing or PI3K inhibition (LY294002) reverses these effects, confirming its role in fibrosis progression (Yu et al., 2025).

### 3.7 FOXO3a

AURKA promotes keloid fibroblast proliferation and migration by forming a positive feedback loop with FOXO3a that activates AKT signaling. This reciprocal regulation enhances fibroblast growth and motility, driving invasive and hyperplastic phenotypes. Beyond its kinase activity, AURKA also acts as a transcription factor, underscoring its multifaceted role in keloid formation and recurrence (Chu et al., 2024).

### 3.8 Zyxin

Zyxin, markedly upregulated in fibrotic fibroblasts, promotes skin fibrosis by activating the FAK/PI3K/AKT and TGF-β pathways through focal adhesion and integrin signaling. This activation enhances fibroblast migration, collagen synthesis, and ECM remodeling, driving skin thickening and collagen deposition in conditions such as systemic sclerosis, keloids, and localized scleroderma (Xu et al., 2023; Huang Y. et al., 2023).

### 3.9 Epac

Epac1 inhibition with ESI-09 suppresses keloid fibroblast proliferation, migration, collagen synthesis, and fibrosis-related marker expression by reducing Akt phosphorylation, while concurrently promoting apoptosis, highlighting Epac1/Akt signaling as a potential therapeutic target in keloid fibrosis (Lv et al., 2021a).

### 3.10 p75NTR

Silencing p75NTR in hypertrophic scar fibroblasts attenuates proliferation, migration, and ECM deposition by enhancing autophagy through inhibition of the PI3K/Akt/mTOR pathway. Increased autophagic activity suppresses fibroblast function, while PI3K agonists or autophagy inhibitors reverse these effects, confirming p75NTR as a regulator of fibrosis via autophagy modulation (Shi et al., 2020; Shi et al., 2021; Dai et al., 2019).

### 3.11 Heat shock protein 90

Hsp90 regulates TGF-β-driven collagen synthesis in dermal fibroblasts by modulating Smad2/3 and Akt signaling. Inhibition with 17AAG suppresses Smad2/3 and Akt phosphorylation, reducing collagen production, whereas Hsp90 overexpression enhances these pathways, underscoring its central role in fibrotic responses (Lee et al., 2016).

### 3.12 Interleukin-10

Interleukin-10 (IL-10) exerts antifibrotic effects by activating the PI3K/AKT and STAT3 signaling pathways, which play key roles in regulating fibroblast behavior and ECM synthesis. Upon binding to its receptor on fibroblasts, IL-10 induces phosphorylation of AKT and STAT3, leading to the downregulation of fibrosis-associated genes such as collagen types I and III and α-SMA. This mechanism helps prevent fibroblast-to-myofibroblast differentiation and limits ECM accumulation, thereby reducing the risk of excessive scar formation (Shi et al., 2014).

### 3.13 c-Met

Phosphorylated c-Met promotes keloid pathogenesis by activating ERK, PI3K, and AKT signaling, which enhances fibroblast proliferation, migration, invasion, and collagen synthesis, thereby driving abnormal wound healing and excessive scar formation (Jin, , 2014).

### 3.14 Endoglin

Endoglin haploinsufficiency enhances Akt signaling, leading to increased fibroblast proliferation, migration, and apoptosis resistance, thereby prolonging inflammation and fibrosis during wound repair. Conversely, endoglin overexpression suppresses Akt activation, indicating its role as a negative regulator of fibroblast accumulation via the PI3K/Akt pathway (Pericacho et al., 2013).

### 3.15 Neuregulin

NRG1 promotes fibrosis in hypertrophic scar fibroblasts by inducing CTGF expression via HER2/HER3 receptor signaling. This effect is mediated through PI3K and Src pathways, as their inhibition suppresses CTGF induction, highlighting NRG1-driven signaling as a key contributor to ECM production and scar formation (Kim et al., 2012).

### 3.16 TGF-β

Silencing fibronectin extra domain B (EDB) suppresses TGF-β1-induced fibroblast proliferation and collagen synthesis by inhibiting Smad2/3, AKT, and ERK phosphorylation. These findings indicate that EDB promotes keloid progression through coordinated activation of TGF-β/Smad and non-Smad (AKT/ERK) pathways, highlighting its potential as a therapeutic target against excessive fibrosis (Cui et al., 2020).

### 3.17 Exosomes

M2 macrophage-derived exosomes promote hypertrophic scar formation by delivering CXCL2, which activates CXCR7-mediated mTOR signaling to induce fibroblast autophagy. This pathway enhances fibroblast proliferation, migration, and collagen synthesis, identifying the CXCL2/CXCR7/mTOR axis as a key mechanism linking macrophage-derived exosomes to fibrotic remodeling (Shi et al., 2024). Exosomes from hypoxia-induced macrophages deliver miR-26b-5p to keloid fibroblasts, where it suppresses PTEN and activates PI3K/AKT signaling. This activation enhances fibroblast proliferation, migration, invasion, and EMT, thereby promoting fibrosis and keloid progression via the exosomal miR-26b-5p/PTEN/PI3K/AKT axis (Dai et al., 2024).

### 3.18 miRNAs

MicroRNAs (miRNAs) are short non-coding RNAs (19–24 nucleotides) that regulate gene expression post-transcriptionally by binding to complementary sequences in target mRNA 3′-UTRs, leading to translational repression or mRNA degradation. Through this mechanism, individual miRNAs modulate multiple genes and are essential for controlling cell proliferation, differentiation, apoptosis, and immune responses (Su and Han, 2024).

#### 3.18.1 MiR-3606-3p

miR-3606-3p attenuates skin fibrosis by targeting ITGAV, GAB1, and TGFBR2, thereby suppressing integrin/FAK, AKT/ERK, and TGF-β/SMAD2/3 signaling. This coordinated inhibition reduces fibroblast proliferation, migration, inflammation, and ECM deposition, highlighting miR-3606-3p as a multifaceted regulator of fibrotic processes in keloids and systemic sclerosis (Chen Y. et al., 2024).

#### 3.18.2 MiR-203a-3p

miR-203a-3p suppresses hypertrophic scar formation by directly targeting PIK3CA, thereby inhibiting PI3K/AKT/mTOR signaling. This downregulation attenuates fibroblast proliferation, migration, collagen synthesis, and myofibroblast differentiation. *In vivo*, miR-203a-3p administration improves scar architecture and reduces collagen accumulation, while PIK3CA co-overexpression reverses these effects, confirming its role in miR-203a-3p–mediated antifibrotic activity (Zhao et al., 2024).

#### 3.18.3 MiR-1-3p and miR-214-5p

TM4SF1 enhances keloid fibroblast proliferation and migration by activating AKT and ERK1/2 signaling. Its expression is suppressed by miR-1-3p and miR-214-5p, whose downregulation in keloids leads to TM4SF1 overexpression and sustained fibroblast activation. Restoration of these miRNAs inhibits TM4SF1 and downstream signaling, underscoring their therapeutic potential in attenuating keloid fibrosis (Xu M. et al., 2020).

#### 3.18.4 MiR-486-5p

MiR-486-5p attenuates hypertrophic scar fibrosis by targeting IGF1 and suppressing the IGF1/PI3K/AKT pathway. Its upregulation reduces collagen I/III and α-SMA expression, inhibits fibroblast proliferation, migration, and invasion, and promotes apoptosis, highlighting miR-486-5p as a potential antifibrotic regulator in hypertrophic scar formation (Xiao, 2021).

#### 3.18.5 MiR-130a

MiR-130a promotes hypertrophic scar progression by targeting and suppressing CYLD, a negative regulator of fibrotic signaling. CYLD downregulation activates the Akt pathway, enhancing fibroblast proliferation and elevating collagen I/III and α-SMA expression, leading to excessive ECM accumulation. Restoring CYLD expression reverses these effects, confirming the profibrotic role of the miR-130a/CYLD–Akt axis (Zhang J. et al., 2019).

#### 3.18.6 MicroRNA-205-5p

MicroRNA-205-5p exerts antifibrotic effects in hypertrophic scars by directly targeting Smad2 and suppressing the PI3K/AKT pathway. Its downregulation in HSFs leads to increased Smad2 expression and ECM synthesis, whereas miR-205-5p restoration reduces collagen I/III and α-SMA levels, highlighting its dual regulatory role in TGF-β and PI3K/AKT signaling and its therapeutic potential in scar modulation (Qi et al., 2019).

#### 3.18.7 MiR-188-5p

MiR-188-5p suppresses keloid fibroblast proliferation, migration, and invasion by inhibiting the PI3K/Akt pathway and downstream MMP-2 and MMP-9 expression. Its reduced expression activates this signaling cascade, promoting fibroblast aggressiveness, whereas miR-188-5p upregulation attenuates the PI3K/Akt/MMP axis, underscoring its antifibrotic role in keloid pathogenesis (Zhu et al., 2019).

#### 3.18.8 MicroRNA-152-5p

MicroRNA-152-5p inhibits keloid fibroblast proliferation and migration while promoting apoptosis by directly targeting Smad3. Through Smad3 suppression, it blocks downstream Akt activation, thereby attenuating fibroblast survival and motility. These findings identify miR-152-5p as a negative regulator of fibrosis via the Smad3–Akt signaling axis and a potential therapeutic target for keloid management (Pang et al., 2019).

#### 3.18.9 MiR-155

MiR-155 mitigates hypertrophic scar formation by targeting HIF-1α and suppressing PI3K/AKT signaling. This inhibition decreases fibroblast proliferation and collagen synthesis, thereby reducing ECM accumulation and fibrotic remodeling, underscoring miR-155’s antifibrotic role in scar pathogenesis (Wu et al., 2018).

#### 3.18.10 MiR-181a

MiR-181a promotes keloid fibroblast proliferation and survival by directly targeting PHLPP2, a negative regulator of AKT signaling. Its overexpression suppresses PHLPP2, leading to enhanced AKT activation, increased DNA synthesis, and reduced apoptosis, highlighting the miR-181a/PHLPP2/AKT axis as a key driver of keloid fibroblast hyperproliferation (Rang et al., 2016).

#### 3.18.11 MiR-21

MiR-21-5p enhances EMT and stem-like phenotypes in keloid keratinocytes by targeting PTEN and activating AKT signaling. Its upregulation reduces E-cadherin and increases vimentin, CD44, and ALDH1 expression, promoting invasiveness and self-renewal (Yan et al., 2016). TGF-β1 promotes fibroblast proliferation and myofibroblast differentiation by inducing miR-21 expression and suppressing PTEN, leading to AKT pathway activation. This signaling cascade increases α-SMA and decreases E-cadherin levels, reinforcing the fibrogenic phenotype (Liu et al., 2016). Furthermore, miR-21 regulates keloid fibroblast proliferation and survival by suppressing PTEN and activating the AKT pathway. Its overexpression enhances DNA synthesis and cell growth, while inhibition restores PTEN expression, reduces AKT activation, and promotes apoptosis, underscoring the miR-21/PTEN/AKT axis as a critical driver of keloid formation and a potential therapeutic target (Liu et al., 2014). Moreover, miR-21 promotes fibroblast proliferation and survival by binding to the 3′-UTR of PTEN mRNA and suppressing its expression, thereby activating the PI3K/AKT pathway. This activation upregulates hTERT, enhancing cell growth and resistance to apoptosis (Zhu et al., 2014).

#### 3.18.12 miR-143-3p

**FIGURE 2:**
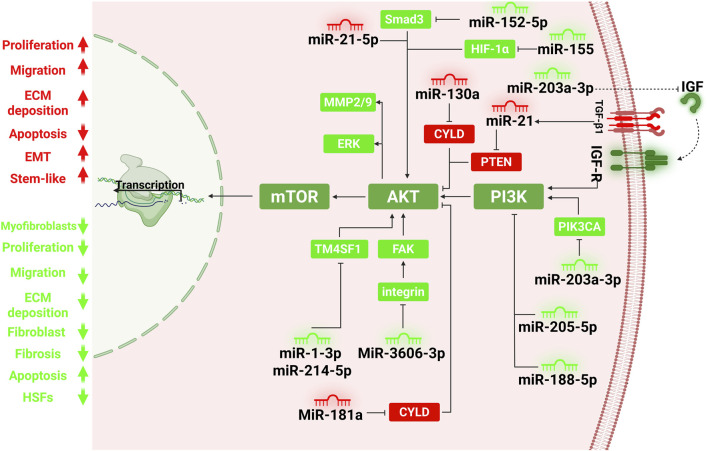
MicroRNA-mediated regulation of the PI3K/AKT/mTOR pathway in fibroblast activation and hypertrophic/keloid scar formation. This figure illustrates the complex post-transcriptional control of the PI3K/AKT/mTOR signaling axis by miRNAs in fibroblasts and scar tissue. miRNAs act as either pro-fibrotic (red) or anti-fibrotic (green) regulators, modulating main signaling nodes such as PTEN, PI3K, AKT, and mTOR, thereby influencing fibroblast proliferation, migration, ECM deposition, and resistance to apoptosis. Pro-fibrotic miRNAs, including miR-21, miR-130a, miR-181a, and miR-26b-5p, promote fibrosis by inhibiting negative regulators like PTEN and CYLD, resulting in unchecked PI3K/AKT activation. These changes enhance collagen production, epithelial-to-mesenchymal transition (EMT), and fibroblast survival. For example, miR-21 is upregulated by TGF-β1 and directly suppresses PTEN, activating AKT and mTOR pathways. Conversely, anti-fibrotic miRNAs, such as miR-1-3p, miR-214-5p, miR-203a-3p, miR-205-5p, miR-152-5p, miR-155, and miR-188-5p, suppress scar formation by targeting members of the PI3K/AKT/mTOR axis or its activators (e.g., Smad3, PIK3CA, HIF-1α, IGF1, TM4SF1). These miRNAs inhibit fibroblast activation, migration, and collagen deposition while promoting apoptosis. Exosome-derived miRNAs from hypoxia-induced or M2 macrophages, such as miR-26b-5p, further contribute to fibrogenesis by modulating recipient fibroblast signaling, particularly via PTEN/AKT or CXCL2/mTOR axes. This integrated network demonstrates the pivotal role of miRNAs in fine-tuning fibroblast behavior and scarring outcomes, positioning them as promising therapeutic targets in hypertrophic and keloid scar management.

MicroRNA-143-3p exerts antifibrotic effects in hypertrophic scar fibroblasts by targeting CTGF, leading to reduced collagen I/III and α-SMA expression, decreased proliferation, and increased apoptosis. By downregulating CTGF, miR-143-3p suppresses Akt/mTOR signaling, whereas CTGF overexpression reverses these effects, indicating that the miR-143-3p/CTGF/Akt/mTOR axis is a critical regulatory pathway in HTS pathogenesis and a promising therapeutic target (Mu et al., 2016) (Figure 2).

### 3.19 LncRNAs

**FIGURE 3:**
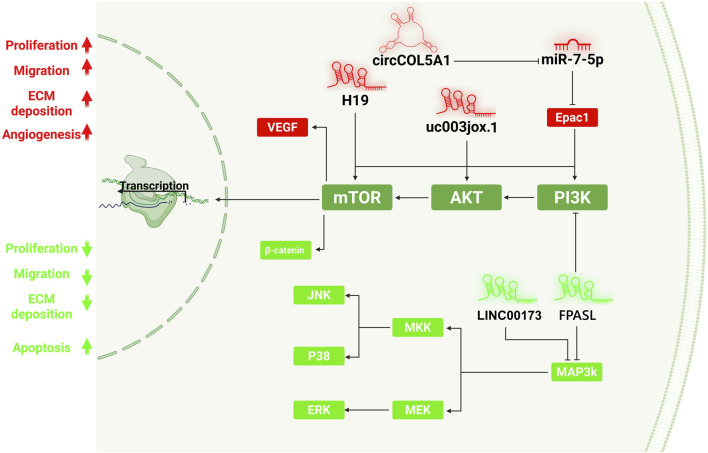
Roles of circRNA and lncRNAs in modulating the PI3K/AKT/mTOR and MAPK pathways during fibroblast activation in keloid and hypertrophic scar formation. This schematic highlights the regulatory influence of lncRNAs and circRNAs on fibroblast behavior in pathological scarring. Activation of PI3K/AKT/mTOR and MAPK signaling cascades drives fibroblast proliferation, ECM deposition, migration, and angiogenesis, hallmarks of keloid and hypertrophic scar formation. Red elements represent pro-fibrotic regulators. LncRNA H19, uc003jox.1, and circCOL5A1 enhance fibroblast activation through direct upregulation of mTOR, AKT, or PI3K signaling. CircCOL5A1 acts as a sponge for miR-7-5p, thereby de-repressing Epac1, a positive regulator of PI3K/AKT. Similarly, uc003jox.1 promotes AKT/mTOR phosphorylation, while H19 also increases VEGF expression to enhance angiogenesis and tissue remodeling. Green components denote anti-fibrotic regulators. LINC00173 promotes fibroblast apoptosis partly by suppressing MAPK activity, whereas FPASL reduces fibroblast proliferation via PI3K/AKT and MAPK pathways, revealing opposite lncRNA-mediated regulation of these signaling cascades in hypertrophic scars. The MAPK cascade, including MEK, ERK, JNK, and P38, is shown as an additional downstream effector of PI3K/AKT involved in cell fate decisions. On the left, the diagram summarizes the functional consequences of pathway activation (red) and suppression (green) on fibroblast phenotype and scar formation. This network underscores how non-coding RNAs cooperatively regulate fibrosis by modulating key intracellular signaling pathways, offering multiple molecular targets for antifibrotic therapies.

Long non-coding RNAs (lncRNAs) are transcripts longer than 200 nucleotides that, despite lacking protein-coding potential, regulate gene expression at multiple levels (HajiEsmailpoor et al., 2024). Synthesized by RNA polymerase II and structurally similar to mRNAs, lncRNAs modulate chromatin organization, transcription, mRNA stability, and translation. They act through diverse mechanisms, including chromatin interaction, transcriptional regulation, and functioning as molecular scaffolds or microRNA sponges, to orchestrate key cellular processes such as proliferation, differentiation, and apoptosis (Su and Han, 2024). The lncRNA uc003jox.1 promotes keloid fibroblast proliferation and invasion by activating the PI3K/AKT/mTOR pathway. Its elevated expression enhances phosphorylation of PI3K, AKT, and mTOR while suppressing apoptosis, whereas uc003jox.1 knockdown upregulates PTEN and attenuates pathway activation, identifying it as a key pro-fibrotic regulator in keloid pathogenesis (Bu et al., 2023). Both LINC00173 and FPASL modulate fibroblast behavior in hypertrophic scars through the PI3K/AKT and MAPK signaling pathways, but in opposite directions. LINC00173 is upregulated in hypertrophic scar fibroblasts and promotes apoptosis, partly associated with reduced p38 MAPK activity. In contrast, FPASL is downregulated and normally acts as a negative regulator of fibroblast proliferation by suppressing AKT, ERK, JNK, and p38 phosphorylation; its loss therefore activates PI3K/AKT and MAPK pathways, driving fibroblast hyperproliferation (Ma et al., 2022; Li Q. et al., 2021). The lncRNA H19 promotes keloid fibroblast proliferation by activating mTOR and VEGF signaling. Its upregulation in keloid tissue enhances cell growth and angiogenic activity, whereas H19 knockdown suppresses proliferation and downregulates mTOR and VEGF expression, identifying H19 as a key pro-fibrotic regulator and potential therapeutic target in keloid pathogenesis (Figure 3) (Zhang et al., 2016).

### 3.20 Circular RNA

Circular RNAs (circRNAs) are a class of non-coding RNAs formed through back-splicing, resulting in a covalently closed loop that confers high stability and resistance to exonuclease degradation. They display tissue- and stage-specific expression patterns and participate in diverse cellular and developmental processes, often functioning as regulators of gene expression (Su and Han, 2024). CircCOL5A1 promotes keloid progression by acting as a ceRNA for miR-7-5p, thereby relieving its repression of Epac1 and activating the PI3K/Akt pathway. This activation enhances fibroblast proliferation, migration, and ECM synthesis while inhibiting apoptosis, identifying the CircCOL5A1/miR-7-5p/Epac1 axis as a key driver of keloid fibrosis and a potential therapeutic target (Lv et al., 2021b).

## 4 Mechanisms associated with PI3K/AKT/mTOR axis in keloids

**FIGURE 4:**
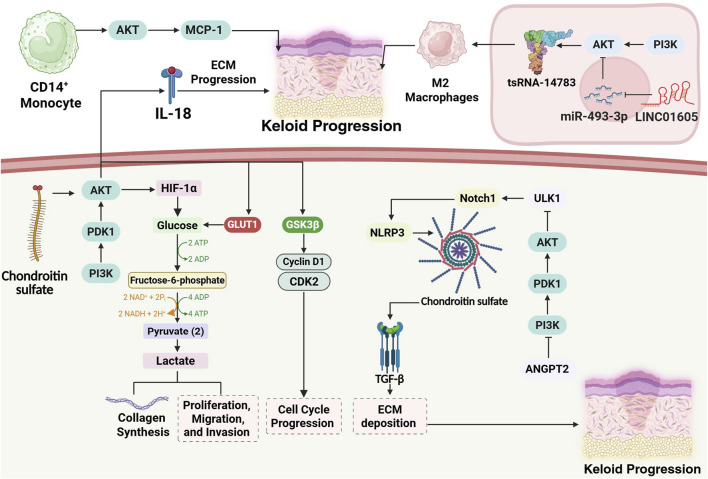
Schematic representation of cellular mechanisms underlying keloid progression, highlighting the central role of the PI3K/AKT/mTOR pathway. In keloid fibroblasts, PI3K/AKT activation, stimulated by chondroitin sulfate and hypoxia-induced HIF-1α, drives a Warburg-like metabolic shift toward glycolysis, increasing lactate production, collagen synthesis, and fibroblast proliferation. PI3K/AKT signaling also promotes ECM deposition via Cyclin D1–CDK2–mediated cell cycle progression and is further reinforced by GSK3β inactivation. Immune dysregulation contributes to keloid pathology through IL-18–driven inflammation, MCP-1–dependent fibroblast activation by CD14^+^ monocytes, and M2 macrophage polarization mediated by tsRNA-14783 and LINC01605-enriched exosomes. Suppressed autophagy, caused by PI3K/AKT-mediated ULK1 inhibition, leads to Notch1 accumulation and NLRP3 inflammasome activation, sustaining TGF-β signaling and fibrosis. Together, these converging pathways orchestrate keloid persistence, invasiveness, and fibrotic remodeling.

Keloid formation results from fibroblast hyperactivity, chronic inflammation, and immune dysregulation. Key mechanisms include excessive collagen I/III production driven by overactive TGF-β/SMAD, JAK/STAT3, and HIF-1α signaling, along with VEGF-induced angiogenesis and NOTCH-mediated fibroblast activation, collectively promoting fibrosis and tissue invasion (Latoni et al., 2024). In this section, we review the cellular mechanisms that are affected by PI3K/Akt/mTOR in keloid formation and progression (Figure 4).

### 4.1 Warburg effect

In keloids, the Warburg effect describes the tendency of fibroblasts to rely on aerobic glycolysis for energy generation despite sufficient oxygen availability, reflecting a metabolic hallmark commonly observed in cancer cells (Su et al., 2022a). Keloid cells adapt to hypoxia through HIF-1α–mediated metabolic reprogramming that shifts energy production toward glycolysis, increasing ATP and lactate levels. This promotes fibroblast proliferation, angiogenesis, and ECM remodeling, enabling keloid persistence and recurrence, and highlighting their tumor-like, quasi-neoplastic nature (Tan et al., 2019). The PI3K/AKT pathway drives glycolytic reprogramming in keloids, enhancing GLUT1, LDHA, and COL1 expression to boost glucose uptake, lactate production, and collagen synthesis. Its inhibition, via PI3K blockade or PGK1 knockdown, reduces glycolysis and PI3K/AKT phosphorylation, thereby suppressing fibroblast proliferation, migration, and invasion (Wang P. et al., 2023). Under hypoxia, PI3K/AKT signaling enhances glycolysis in keloid fibroblasts by upregulating GLUT1 and key enzymes (HK2, PFKFB3, LDHA), increasing ECAR and promoting proliferation, migration, and survival. Inhibition with LY294002 reverses this effect by restoring oxidative phosphorylation and suppressing fibroblast growth. Moreover, PI3K/AKT sustains redox homeostasis and forms a positive feedback loop with HIF-1α, further amplifying glycolytic reprogramming and reinforcing the tumor-like behavior of keloids (Wang Q. et al., 2023). The Akt-GSK3β-Cyclin D1 signaling pathway plays a crucial role in mediating the effects of Warburg effect inhibition in keloid fibroblasts. The inhibition of the Warburg effect by oxamate, a lactate dehydrogenase A (LDHA) inhibitor, decreased Akt expression and reduced phosphorylation of GSK3β (at Ser9), which activated GSK3β. Activated GSK3β promoted the phosphorylation and degradation of Cyclin D1, a key regulator that drives cell cycle progression from G1 to S phase (Su et al., 2022b).

### 4.2 Extracellular matrix formation

The PI3K/AKT/mTOR pathway regulates tumorigenic ECM remodeling by enhancing ECM protein synthesis, activating fibroblasts, stimulating ECM-degrading enzymes, and modulating integrin-mediated adhesion, processes that may contribute to cancer therapy resistance (Klabukov et al., 2024; Shamsan et al., 2024; Li et al., 2025a). Activation of this pathway enhances fibroblast proliferation and survival, promotes the synthesis of key ECM proteins such as collagen and fibronectin, and stimulates the activity of ECM-degrading enzymes like MMPs, which together drive aberrant matrix remodeling. Moreover, PI3K/AKT/mTOR signaling modulates integrin-mediated cell–matrix adhesion and crosstalk with other pathways, including MAPK and TGF-β/Smad (Luo et al., 2017). Chondroitin sulfate (CS) promotes keloid fibroblast proliferation via PI3K/AKT activation, not MAPK/ERK or JNK signaling. CS induces time-dependent AKT phosphorylation through integrin α1–FAK signaling, reducing p21 and activating CDK2 to drive the cell cycle. Blocking integrin α1 or inhibiting PI3K with wortmannin abolishes AKT activation and KF proliferation, confirming the pathway’s key role in this CS-mediated effect (Katayama et al., 2020).

### 4.3 Mechanical properties

Mechanical stress drives hypertrophic scar formation by activating the PI3K/AKT pathway, which promotes fibroblast survival and ECM remodeling. During the proliferative phase of wound healing, mechanical loading activates Akt, inhibiting apoptosis through suppression of pro-apoptotic factors (Bad, p53) and upregulation of Bcl-2. This Akt-dependent anti-apoptotic signaling enhances fibroblast accumulation and collagen deposition, linking mechanotransduction to fibrosis and tumor-like matrix remodeling (Aarabi et al., 2007). Stiffened ECM activates integrin–FAK signaling, which subsequently stimulates the PI3K/AKT pathway, forming a positive feedback loop that enhances cellular invasion. This mechanotransductive mechanism underlies both keloid scar formation and oncomatrix development (Zhao et al., 2025). Static and cyclic strain rapidly induce Akt phosphorylation in murine fibroblasts, enhancing migration and motility through the PI3K/Akt pathway. High-frequency cyclic strain produces stronger Akt activation than static strain, indicating frequency-dependent mechanosensitivity. *In vivo*, mechanical stress on wounded and unwounded skin similarly activates Akt and upregulates α-SMA expression, although inhibition of PI3K/Akt signaling increases apoptosis without reducing scar formation (Paterno et al., 2011). Mechanical pressure significantly downregulates the expression of IGF-1, IGF-1R, and PI3K, leading to decreased activation of the PI3K/AKT pathway in scar tissues. Since PI3K/AKT signaling typically promotes fibroblast proliferation, survival, and collagen synthesis, its inhibition under pressure resulted in reduced fibroblast accumulation and extracellular matrix deposition, thereby improving scar histology (Liu et al., 2019). A subvacuum environment (1/10 atm) promotes wound healing by activating the Ca^2+^-dependent PI3K/AKT pathway. This activation enhances keratinocyte and fibroblast migration without increasing proliferation. Elevated levels of PI3K, p-PI3K, AKT1, and p-AKT1 lead to cytoskeletal depolymerization and increased membrane fluidity, facilitating cell motility. Blocking mechanosensitive Ca^2+^ channels abolished these effects, confirming PI3K/AKT’s role in mechanotransduction. *In vivo*, subvacuum dressings accelerated epithelialization and healing without inducing hypertrophic scarring (Jin et al., 2023).

### 4.4 Immune cells and M2 macrophage polarization

Immune cells play a key role in keloid formation by driving chronic inflammation and promoting fibrosis. Mast cells, macrophages, Tregs, CD8^+^ T cells, dendritic cells, and NK cells contribute to the abnormal wound healing in keloids. These cells release signals that activate fibroblasts, increase collagen production, and suppress normal immune responses, leading to the excessive scarring that defines keloids (Lee et al., 2023). CD14^+^ monocytes from keloid patients enhance MCP-1 secretion, which drives fibroblast proliferation via Akt activation. MCP-1 increases Akt phosphorylation, while inhibition of Akt (LY294002) or neutralization of MCP-1 suppresses this effect. CD14^−^ cells lack this activity, confirming that monocyte-derived MCP-1–Akt signaling is a key promoter of fibroblast overgrowth in keloid pathogenesis (Liao et al., 2010).

The tRNA-derived fragment tsRNA-14783 promotes M2 macrophage polarization in keloids by activating the Wnt and PI3K-Akt pathways. This dual signaling enhances macrophage survival, metabolism, and anti-inflammatory activity, driving a reparative, pro-fibrotic phenotype that fosters tissue remodeling and contributes to keloid progression (Wang and Hu, 2022). Inhibiting M2 macrophage–derived exosome release with GW4869 blocks LINC01605 transfer to dermal fibroblasts, preventing miR-493-3p suppression and AKT1 upregulation. This disruption attenuates AKT/mTOR signaling, reducing fibroblast proliferation, migration, invasion, and collagen synthesis, thereby limiting fibrosis (Zhu et al., 2021).

### 4.5 Autophagy

Autophagy is a key cellular mechanism that clears damaged organelles and proteins to preserve cellular balance, especially during stress conditions (Gómez-Virgilio et al., 2022). In keloid pathology, however, autophagy is disrupted, marked by elevated expression of autophagy-related proteins alongside impaired autophagic flux. This dysfunctional autophagy results in the buildup of pro-fibrotic and pro-inflammatory mediators, including Notch1 and NLRP3, which contribute to abnormal scar formation and persistent tissue fibrosis (Lee et al., 2020). Similarly, in hidradenitis suppurativa (HS), impaired autophagy is associated with defective immune regulation, follicular occlusion, and persistent inflammation. Targeting autophagy pathways may offer therapeutic potential in both conditions by modulating inflammation and abnormal tissue remodeling (Kim et al., 2022). In keloid fibroblasts, excessive PI3K-AKT-mTOR activity suppresses autophagy by inhibiting ULK1, leading to impaired autophagic flux and buildup of undegraded components. Accumulated Notch1 activates the NLRP3 inflammasome and enhances TGF-β secretion, driving persistent inflammation and excessive ECM deposition typical of keloid fibrosis (Kim et al., 2022). Inhibiting ANGPT2 activates autophagy in hypertrophic scars by downregulating the PI3K/AKT/mTOR pathway. Since ANGPT2 normally promotes fibrosis through this signaling cascade, its suppression reduces fibrotic activity and restores autophagic function, helping limit scar formation (Chen et al., 2023).

### 4.6 Epithelial-mesenchymal interactions

The interleukin-18 (IL-18) signaling drives keloid progression by promoting pathological epithelial–mesenchymal interactions between keratinocytes and fibroblasts. Elevated IL-18 enhances collagen and pro-fibrotic cytokine (IL-6, IL-8) secretion, increases caspase-1 activity, and suppresses IL-10. Its expression depends on PI3K/mTOR, MAPK, and Sp1 pathways, making IL-18 a key mediator of fibroblast activation and a potential therapeutic target in keloid scarring (Do et al., 2012).

## 5 Targeting PI3K/AKT/mTOR signaling for keloid therapy

Targeting the PI3K/AKT/mTOR pathway presents a promising therapeutic approach for keloid treatment, given its central role in controlling fibroblast proliferation, migration, survival, and myofibroblast differentiation, processes fundamental to keloid pathogenesis (Kim and Kim, 2024). Keloid treatment involves diverse approaches, including silicone therapy, intralesional injections (corticosteroids, 5-FU, bleomycin), surgery, radiation, laser, and cryotherapy. Emerging systems like microneedles, nanoparticles, liposomes, and exosome-based delivery enhance precision and reduce side effects. Combination therapies are typically more effective due to high recurrence rates (Mishra and Wairkar, 2025). This section reviews the latest treatment targeting the PI3K/AKT/mTOR pathway in keloids (Table 1).

**TABLE 1:** Different treatments targeting PI3K/AKT/mTOR in hypertrophic scars and keloids.

Therapeutic agent	Type of intervention	Cell models used	Experimental model (in vitro/*In Vivo*)	Treatment conditions (dose/Duration)	Molecular targets and pathways	Observed effects (key findings)	Ref.
Synthetic drugs							
Lapatinib	Tyrosine Kinase Inhibitor	Keloid Fibroblasts, Primary Mouse Dermal Fibroblasts (PSFs)	*In vitro* and *in vivo*	2.5, 5, 10, 20 μM; daily for 7–21 days, depending on the model	ErbB1/ErbB2; TGF-β1/Smad2/3, ERK, and AKT signaling	- Reduced fibroblast proliferation and migration - Suppressed ECM protein expression - Inhibited TGF-β1-induced activation - Effective in keloid and SSc models	Wang et al. (2025)
Artesunate (ART)	Drug (Artesunate)	HUVECs, Human skin fibroblasts	*In vivo* (Rabbit ear), *In vitro*	*In vivo*: 3 and 6 mg/mL every 48 h for 2 months *In vitro*: 20 μg/mL for 24 h	PI3K/Akt/mTOR, TGF-β1/Smad, EndMT	- Reduced HS protrusion and thickness -Inhibited fibroblast proliferation and EndMT - Decreased α-SMA, COL1A1, FN1. expression - Improved collagen organization - Reduced ECM deposition	Shang et al. (2025)
Remdesivir (RD)	Small molecule drug (nucleoside analog)	- PSFs (Primary Skin Fibroblasts) - KFs (Keloid Fibroblasts)	- In vivo: BLM-induced skin fibrosis mouse model (C57BL/6J) Keloid xenograft model (nude BALB/c mice) - In vitro: TGF-β1-stimulated fibroblasts	- In vivo: 12.5 µM and 25 µM daily injections for 2–3 weeks - In vitro: 12.5 µM and 25 μM, 24 h treatment	- TGF-β/Smad3 signaling pathway- PI3K/Akt/mTOR pathway	- ↓ Fibroblast proliferation and migration - ↓ ECM synthesis (Collagen I, Fibronectin) - ↓ Autophagy markers (p62, LC3) - ↓ Inflammatory markers (IL-6, MMPs) - ↓ Phosphorylation of Smad3, Akt, PI3K, mTOR - ↓ Skin thickness and collagen deposition - Attenuates both fibrosis and autophagy	Zhang et al. (2024a)
Axitinib (AG-013736)	VEGFR Tyrosine Kinase Inhibitor	Not specified	*In vivo* (Rabbit Ear Hypertrophic Scar Model)	200 µL of 1.25 mg/mL solution, intralesional injection once per week for 2 weeks	VEGF-VEGFR signaling; downstream AKT/p70S6K pathway	- Significantly reduced scar thickness, vascularity, and pliability - Decreased collagen volume fraction and scar elevation index - Suppressed CD31 expression, indicating reduced angiogenesis - Lowered levels of p-AKT, p70S6K, and p-p70S6K - No adverse effects such as allergy, erythema, erosion, or dyspigmentation were observed	Liu et al. (2023)
Sunitinib	Kinase Inhibitor	KFs, NFs, 293T, ADSCs, BMSCs	*In Vitro*	2–6 μM, 24–48 h	Akt/PI3K/mTOR pathway	- Selective cytotoxicity to KFs- Cell cycle arrest at S phase - Induced apoptosis in KFs - Inhibited 3D migration/invasion - Downregulated collagen I and III - Suppressed PI3K, Akt, mTOR expression	Chen et al. (2022a)
UDC-907	Dual Inhibitor (PI3K/Akt/mTOR and HDAC)	Keloid Fibroblasts, HUVECs	*In vitro*, *Ex vivo*, *in vivo* (xenograft)	0.32, 3.2, 32 nM for 5–7 days (*in vitro*); 64 nM injection every 2 days (*in vivo*)	PI3K/Akt/mTOR, HDAC2, TGF-β1, Smad, Erk	- Suppresses KF proliferation - Induces G2/M arrest - Inhibits migration and invasion- Reduces collagen (I/III) and TGF-β1 - Decreases angiogenesis - Effective in vivo and ex vivo	Tu et al. (2019)
SI-027	mTOR kinase inhibitor (2nd generation)	Primary human keloid keratinocytes	*In vitro*	100 nmol/L for 2–72 h	mTORC1 and mTORC2; inhibits S6K1, 4EBP1, AKT (Ser-473) phosphorylation	- Inhibits keratinocyte proliferation (MTT, BrdU, [H3] incorporation) - Reduces cell migration (track motility, transwell assay) - Disrupts mTORC1 and mTORC2 complex assembly - No induction of cell death - More potent than rapamycin	Chen et al. (2019)
Linagliptin	Dpp4 Inhibitor	Hypertrophic scar-derived fibroblasts (HSF)	*In Vitro*	200 μmol/L; 24–48 h	IGF/Akt/mTOR pathway; Dpp4	- ↓ Collagen I and III expression - ↓ α-SMA expression (myofibroblast marker) - ↓ Dpp4 protein levels - ↓ Phosphorylation of IGF, Akt, and mTOR - ↓ Cell proliferation and migration - Inhibited fibroblast-to-myofibroblast transdifferentiation	Li et al. (2019)
Fingolimod (dFTY720)	Immunomodulatory/Antifibrotic agent	Hypertrophic Scar Fibroblasts (HSFs), Normal Fibroblasts (NFs)	*In Vitro* and *In Vivo* (Rabbit Ear Model)	*In vitro*: 0–20 μmol/L for 6, 12, 24, 48 h *In vivo*: 5 mg/mL subcutaneous injection for 2 weeks after 21-day scar formation	S1PR5, PI3K/Akt/mTOR/p70S6K signaling pathway	- Induces G0/G1 cell cycle arrest - Promotes intrinsic apoptosis (↑Bax, cleaved PARP, caspase 3/9; ↓Bcl-xL) - Inhibits migration and contraction- Reduces α-SMA, collagen I/III expression - Suppresses Akt/mTOR/p70S6K signaling - Reduces collagen deposition and macrophage infiltration in vivo - Minimal effect on normal fibroblasts	Shi et al. (2017)
17-AAG (17-allylaminodemethoxygeldanamycin)	HSP90 Inhibitor (Small Molecule Drug)	Keloid Fibroblasts, Normal Human Dermal Fibroblasts	*In Vitro*	0, 5, 10, 20 µM for 48 h	HSP90 → Akt Pathway	- Induces apoptosis in keloid fibroblasts (dose-dependent) - Decreases proliferation and metabolic activity - Suppresses Akt protein expression - Reduces migration velocity and distance in wound healing assays - Decreases the directionality of fibroblast migration	Yun et al. (2015)
U-0063794	mTORC1/2 inhibitor (ATP-competitive)	Keloid fibroblasts, Extra-lesional fibroblasts (ELFs)	*In vitro*, *Ex vivo*	2.5–20 μM (24–48 h); *Ex vivo*: 10 μM (3–28 days)	PI3K/Akt/mTOR, pAkt-Ser473, mTORC1, mTORC2	- Inhibited pAkt-S473, p-mTOR, and downstream signaling - Reduced KF proliferation, attachment, migration, and invasion - Induced apoptosis selectively in KFs - Suppressed ECM proteins: collagen I, fibronectin, α-SMA - Caused shrinkage and apoptosis in keloid tissue - Depleted angiogenic markers (CD31, CD34)	Syed et al. (2013)
U-0068650	mTORC1/2 inhibitor (ATP-competitive)	KFs, ELFs	*In vitro*, *Ex vivo*	2.5–20 μM (24–48 h); *Ex vivo*: 10 μM (3–28 days)	PI3K/Akt/mTOR, pAkt-Ser473, mTORC1, mTORC2	- Similar but greater efficacy than KU-0063794 - Strong inhibition of KF activity at low doses - Induced more sustained apoptosis and tissue regression - Downregulated ECM and cell cycle proteins - Potent anti-angiogenic and anti-fibrotic effects	Syed et al. (2013)
P529	Dual mTORC1/mTORC2 Inhibitor	Keloid Fibroblasts, Normal Fibroblasts (NF)	*In vitro*, *Ex vivo* (Organ Culture)	*In vitro*: 5, 10, 20, 40 ng/mL; 24–48 h *Ex vivo*: 20 ng/mL, up to 4 weeks	PI3K/Akt/mTOR pathway Inhibits p-Akt (Ser473), p-mTOR, pS6, 4E-BP1	- Inhibits cell proliferation, migration, and invasion - Downregulates ECM proteins (collagen I, fibronectin, α-SMA) - Induces apoptosis (early and late) - Reduces angiogenesis (↓ CD31+, CD34+ cells) - Reduces tissue volume and epidermal thickness - Suppresses PCNA and Cyclin D expression	Syed et al. (2012)
LSKL Peptide	TSP-1 Antagonist Peptide	HSFs (Hypertrophic Scar Fibroblasts), NSFs (Normal Skin Fibroblasts)	Both *In vitro* and *In vivo* (Rat Tail HTS Model)	*In vitro*: 0–100 μM for 24–120 h *In vivo*: 3 mg/kg, intradermally 3x/week for 4 weeks	Inhibits TSP-1, suppresses TGF-β activation↓ PI3K/AKT/mTOR phosphorylationNo significant effect on Smad2/3 or MEK/ERK	- Downregulates Collagen I and α-SMA expression (dose- and time-dependent) - Inhibits fibroblast proliferation and migration - Induces apoptosis in HSFs - Reduces collagen gel contraction - Alleviates scar thickness and collagen disorganization in vivo - Lowers scar index in rat model	Xu et al. (2020c)
Hydrogels							
A-peptide hydrogel	Peptide hydrogel	HSFs, Peritoneal Macrophages	*In vitro* and *In vivo*	10% (*in vivo*), 1% (*in vitro*), 18–35 days *in vivo*	TGF-β, PI3K/Akt, Smad2/3	- Inhibits M2 macrophage polarization - Reduces fibroblast activation - Decreases TGF-β levels - Suppresses ECM deposition - Promotes scarless wound healing	Li et al. (2025b)
GD/CS/CS/β-GP Hydrogel	Thermosensitive bio-hydrogel	3T3, Hs68	*In vitro* and *in vivo*	Injected hydrogel, sustained release over several days	SMAD, P38 MAPK, PI3K	- Inhibits fibroblast overproliferation - Reduces postoperative adhesions - Downregulates fibrosis-related pathways (SMAD, PI3K) - Suppresses inflammatory signaling (P38 MAPK) - Promotes wound healing and tissue regeneration	Liang et al. (2025)
Liposomes							
aclitaxel–cholesterol-loaded liposomes (PTXL)	Drug-loaded liposome	HKFs (human keloid fibroblasts)	*In vitro* and *in vivo*	*In vitro*: 0.1 μg/mL for 24–48h; *In vivo*: 0.05 µg/100 mm^3^, every 7 days × 4	PI3K/AKT/GSK3β, TNF-α, IL-6, TGF-β	- Enhanced stability and uptake - Stronger proliferation inhibition - Induced apoptosis and G2/M arrest - Reduced migration/invasion - Lowered α-SMA, collagen I	Wang et al. (2019)
Exosomes							
ADSCEs (Normoxic ADSC Exosomes)	Exosome-based stem cell therapy	Keloid Fibroblasts	*In vitro* and *in vivo* (mouse keloid graft model)	100 μg/mL for 24 h (*in vitro*); 50 µL every other day post-op day 7 to day 21 (*in vivo*)	PI3K/AKT/mTOR signaling pathway	- Inhibited proliferation and migration of KFs - Decreased collagen deposition (COL1, COL3, α-SMA) - Suppressed inflammatory cytokines (TGF-β1, TNF-α, IL-6) - Promoted mitochondrial autophagy and improved mitochondrial morphology	Liu et al. (2024)
Stem cells							
Adipose-Derived Stem Cells (ADSCs)	Stem cell-based therapy	Human keloid-derived fibroblasts	*In Vitro*	48 h with ADSC-conditioned medium	ITGA2, PI3K/Akt signaling pathway	- Inhibits fibroblast proliferation - Promotes fibroblast apoptosis - Downregulates ITGA2 expression- Disrupts fibrotic signaling via PI3K/Akt pathway	Chen et al. (2022b)
Microneedles							
Gla-loaded Microneedle (Gla-MN)	Microneedle delivery system	Not specified (used in rabbit model)	*In Vivo* (Rabbit ear keloid model)	Inserted into scar tissue once/day for 12 days	PI3K/Akt and TGF-β1/α-SMA	- Greater scar reduction than Gla alone - Higher apoptosis induction (↑ Bax, ↑ cleaved caspase-3, ↓ Bcl-2) - Greater inhibition of collagen synthesis - Improved skin penetration and drug release (76.9% in 24 h)	Guo et al. (2024)
Nanoparticles							
Se@SiO2 NPs	Nanoparticle (antioxidant delivery)	Human Skin Fibroblasts (HSFs)	*In vivo* (rat), *In vitro* (HSFs)	*In vivo*: 1 mg/mL topical application; *In vitro*: 30 μg/mL for 24 h	ROS reduction, PI3K/Akt pathway	- Accelerated wound closure - Reduced collagen and fibronectin expression - Decreased fibroblast apoptosis - Suppressed myofibroblast activation - Increased p-Akt signaling - Lowered ROS levels - High biosafety, no organ toxicity	Yang et al. (2022)
Laser and radiation therapy							
High-energy Laser (20 J/cm^2^)	Photoelectric Therapy	HSF (Human Skin Fibroblast)	*In Vitro*	20 J/cm^2^, 20 h	↑ miR-206, ↓ mTOR, ↓ cyclin D1, ↓ Bcl-2, ↑ Bax, ↑ MMP-9	- Inhibits cell proliferation - Promotes apoptosis - Upregulates pro-apoptotic Bax - Downregulates anti-apoptotic Bcl-2 - Enhances MMP-9 (ECM degradation) - Decreases mTOR signaling	Zhang et al. (2020)
Low-energy Laser (10 J/cm^2^)	Photoelectric Therapy	HSF (Human Skin Fibroblast)	*In Vitro*	10 J/cm^2^, 20 h	↑ miR-206 (moderate), ↓ mTOR (moderate), slight ↑ Bax, ↓ cyclin D1	- Slight inhibition of proliferation - Mild increase in apoptosis - Intermediate effects compared to the high-energy group	Zhang et al. (2020)
Electron Beam (EB) Irradiation	Radiation Therapy	Keloid fibroblasts	*In Vitro*	20 Gy, single dose, effects observed up to 3 days post-irradiation	miR-21-5p ↓, PTEN ↑, p-AKT ↓, LC3B-II ↓, Beclin-1 ↓	- Induces apoptosis in keloid fibroblasts - Inhibits autophagy - Suppresses cell migration - Downregulates miR-21-5p expression- Activates PTEN/AKT signaling axis	Yan et al. (2020)
Natural products							
WuFuYin (WFY)	Traditional Chinese Medicine	Not specified	In silico + network pharmacology	Not applicable	PI3K-Akt, ErbB, Rap, FoxO pathways; hub genes: CDK2, GSK3B, PIK3CG	- Modulates multiple signal pathways - Reduces inflammation and fibrosis - Targets key genes involved in cell proliferation and apoptosis	Zhang et al. (2024b)
Shikonin (SHI)	Natural compound (naphthoquinone)	Hypertrophic scar-derived fibroblasts (HSFs)	*In vitro* and *in vivo* (porcine model)	*In vitro:* 1.25–2 μM for 24 h (optimal 1.75 μM) *In vivo:* Administered over 0–8 weeks	AMPK/mTOR signaling pathway LC3, Beclin1, P62, ULK1	- Induces autophagy in HSFs - Inhibits HSF proliferation - Reduces collagen deposition - Promotes expression of autophagy markers (LC3-II, Beclin1) - Reduces expression of P62 - Modulates AMPK (↑p-AMPK) and mTOR (↓p-mTOR) - Enhances scar repair in vivo	Zhang et al. (2023b)
Silibinin	Natural flavonoid (phytochemical)	Human dermal fibroblasts (HDFs), Keloid-derived fibroblasts (KFs)	*In vitro*	100–200 μM for 24 h	- mTOR signaling pathway (including p70S6K, S6, 4E-BP1) - TGF-β1-induced signaling	- Reduced mRNA expression of COL1A1 and COL3A1 in dose-dependent manner - Decreased phosphorylation of mTOR and downstream effectors - No cytotoxic effects at doses ≤200 μM - Suppressed TGF-β1-induced collagen production - Suggests anti-fibrotic and scar-reducing potential	Choi et al. (2023)
Melatonin	Hormone/Small molecule	HSFs (Hypertrophic Scar Fibroblasts), NFs (Normal Fibroblasts)	Both *In Vitro* and *In Vivo* (Rabbit ear model)	*In vitro*: 25–400 μM, typically 200 µM for 24 h *In vivo*: 5 mg/kg/day IP injection for 21 days	MT2 receptor → PI3K/Akt/mTOR inhibition → Enhanced autophagy	- ↓ Collagen I/III, α-SMA - ↓ Migration and contraction of HSFs - ↑ LC3-II/↓ p62 (autophagic flux)- ↓ PI3K/Akt/mTOR phosphorylation - Improved scar morphology and reduced SEI	Dong et al. (2024)
Melatonin	Hormone	Keloid Fibroblasts, Normal Fibroblasts (NFs), Normal Scar Fibroblasts (NSFs)	*In Vitro*	0.5–2 mmol/L for 24–96 h	MT2 → ↓cAMP/PKA → ↓BRAF/Erk; ↓Smad2/3 phosphorylation	- Induced KF apoptosis - Inhibited KF proliferation - Inhibited migration, invasion, and contraction- Reduced collagen I/III expression - No effect on NFs	Huang et al. (2023b)
Glabridin (Gla)	Natural flavonoid (from *Glycyrrhiza glabra*)	Human Keloid Fibroblasts	*In vitro*	5–100 μg/mL for 12, 24, 48 h	PI3K/Akt and TGF-β1/SMAD signaling	- Inhibits HKF proliferation (dose- and time-dependent) - Induces apoptosis (↑ Bax, c-caspase-3/-9; ↓ Bcl-2) - Reduces mitochondrial membrane potential - Suppresses PI3K and Akt phosphorylation - Downregulates α-SMA, COL1A1, and COL3A1 expression - Inhibits TGF-β1, TGFBR1/2, SMAD2/3 phosphorylation and nuclear translocation	Zhang et al. (2022)
Glabridin (Gla)	Natural flavonoid (from *Glycyrrhiza glabra*)	—	*In vivo* (Rabbit ear hyperplastic scar model)	0.5%, 1%, 2% topical application daily for 21 days (starting day 14 post-wounding)	PI3K/Akt and TGF-β1/SMAD signaling	- Reduces scar thickness and collagen deposition - Improves wound healing - Decreases inflammatory markers (IL-1β, IL-6, TGF-β1) - Flattens scar tissue and improves histopathology - Lowers Masson and H&E pathological scores	Zhang et al. (2022)
Panax Notoginseng Saponins (PNS)	Traditional Chinese Medicine/Natural Compound	HSFs (Hypertrophic Scar Fibroblasts), NHDFs (Normal Human Dermal Fibroblasts)	*In Vitro*	0, 200, 300, 400 μg/mL for 24 h; IC50 = 475 μg/mL (HSFs), 620 μg/mL (NHDFs)	TRPM7, PI3K/AKT pathway	- Suppresses TRPM7 expression- Inhibits PI3K/AKT signaling - Reduces fibroblast proliferation - Promotes apoptosis of HSFs - Decreases collagen synthesis and ECM deposition - Alleviates hypertrophic scar formation	Zhi et al. (2021)
Wubeizi Ointment	Traditional Chinese Medicine	Human keloid-derived fibroblasts	*In vivo* (nude mice) and *In vitro* (cell culture)	*In vivo*: 3% and 5% ointment, 0.5 g/10 mm^2^, 3× daily for 30 days *In vitro*: 0.5 mg/mL for 12–48 h	mTOR signaling pathway (PI3K/Akt/mTOR/PTEN)	- ↓ Akt1 and mTOR mRNA and protein levels - ↑ PTEN mRNA and protein levels - No significant change in PI3K - ↓ Keloid size in mice (dose-dependent) - ↓ Fibroblast proliferation (MTT assay) - ↑ Apoptosis (flow cytometry) - Effects attenuated by IGF-1, an mTOR agonist	Tang et al. (2020a)
Tetramethylpyrazine (TMP)	Traditional Chinese Medicine Component	HS-derived fibroblasts (HFs)	*In Vitro*	1, 5, 10, 20, 40 μM; 24–72 h	PI3K/AKT signaling pathway, mitochondrial apoptotic pathway (Bcl-2/Bax, Caspase-3)	- Inhibited expression of Col I, Col III, and α-SMA (fibrosis markers) - Induced morphological changes and apoptosis in HFs - Arrested cell cycle at G0/G1 phase - Downregulated AKT phosphorylation (p-AKT) - Activated pro-apoptotic proteins Bax and cleaved Caspase-3 - Suppressed anti-apoptotic Bcl-2 expression	Wu et al. (2020)
Galla chinensis ointment	Traditional Chinese Medicine	Keloid fibroblasts	*In vitro*	0.5 mg/mL for 24 and 48 h	miR-21/PTEN/PI3K/Akt/mTOR signaling pathway	- ↓ miR-21 expression - ↑ PTEN expression - ↓ p-Akt and p-mTOR - No change in PI3K expression - Inhibited fibroblast proliferation	Tang et al. (2020b)
Galla chinensis ointment	Traditional Chinese Medicine	N/A (keloid tissue)	*In vivo* (nude mouse model)	5% ointment, 0.5 g/10 mm^2^, applied 3 times/day for 30 days	miR-21/PTEN/PI3K/Akt/mTOR signaling pathway	- ↓ miR-21 expression - ↑ PTEN expression - ↓ Akt and mTOR protein expression - Reduced keloid tissue size	Tang et al. (2020b)
Pentoxifylline (PTX)	Pharmacological (Methylxanthine derivative)	Hypertrophic scar fibroblasts (HSFs), normal dermal fibroblasts (NFs)	*In vitro* and *in vivo* (Bleomycin-induced mouse model)	*In vitro*: 0, 0.25, 0.5, 1, and 2 mg/mL for 24–48 h *In vivo*: 25 mg/kg and 50 mg/kg, local injections for 8 weeks	PI3K/Akt/FoxO/p27^Kip1^ pathway	- ↓ Col1, Col3, α-SMA expression (dose- and time-dependent) - ↓ Fibroblast proliferation (via G1 arrest) - ↑ p27^Kip1^ expression - ↓ Akt phosphorylation, ↑ FoxO1 activation - ↓ Dermal thickening and collagen deposition in vivo - No significant induction of apoptosis	Yang et al. (2019)
Quercetin	Natural compound	Keloid fibroblasts	*In Vitro*	20/40/80 μmol/L for 24 h	PI3K/Akt pathway, HIF-1α	- Dose-dependent apoptosis - Reduced p-Akt and HIF-1α - Sensitizes keloid fibroblasts to IR	Si et al. (2018a)
Quercetin + Ionizing Radiation (IR)	Combination therapy	Keloid fibroblasts	*In Vitro*	20 Gy IR + 40 μmol/L Quercetin for 24 h	PI3K/Akt pathway, HIF-1α	- Synergistic effect increases apoptosis - Sensitization peaks at 40 μmol/L Quercetin	Si et al. (2018a)
Acteoside	Herbal Glycoside	NHDF	*In Vitro*	6.25–100 μM; 4–24 h	MT1-MMP, proMMP-2 via PI3K pathway	- Enhances proMMP-2 activation - Increases MT1-MMP expression - No cytotoxicity observed	Si et al. (2018b)
Gallic Acid (GA)	Plant-derived polyphenol	Keloid Fibroblasts	*In vitro* and *Ex vivo*	*In vitro*: 25–200 μM for 12–72 h (optimal: 100 μM) *Ex vivo*: 100 μM for 3–8 days	AKT/ERK signaling MMPs/TIMPs VEGF/VEGFR	- Inhibits KF proliferation - Induces G1 cell cycle arrest - Promotes apoptosis - Suppresses migration and invasion - Downregulates MMP-1/3, upregulates TIMP-1- Inhibits angiogenesis via VEGF/VEGFR suppression	Wang et al. (2018)
Naringin	Natural flavonoid (Traditional Chinese medicine)	Hypertrophic scar fibroblasts (HTSFs), Human keloid fibroblasts	*In vitro*	IC_50_ for HTSFs: 49.75 μmol/L; for HKFs: 38.63 μmol/L; Exposure time: 24 h	Akt kinase (Aktp-Ser473/Thr308); PI3K/Akt signaling pathway	- Inhibits fibroblast proliferation (dose-dependent) - Induces cell cycle arrest - Promotes apoptosis - Suppresses fibroblast motility - Inhibits Akt kinase activity and phosphorylation - Downregulates Akt downstream targets	Song et al. (2018)
Resveratrol	Non-flavonoid polyphenolic compound (phytoalexin)	Pathological scar fibroblasts	*In vitro*	Different concentrations: exact doses and times are not specified clearly in the provided summary	mTOR/70S6K signaling pathway	- Reduced proliferation of pathological scar fibroblasts	Tang et al. (2017b)
Emodin	Herbal-derived compound (Traditional Chinese medicine)	Hypertrophic Scar Fibroblasts (HSFs), THP-1 human monocytic cells, Normal fibroblasts (NFs)	Both *in vivo* (C57BL/6 mice model) and *in vitro* (cell culture assays)	*In vivo*: 10 mg/kg/day (intraperitoneally) from day 4–14 post-incision *In vitro*: 20, 40, 80, 120 μg/mL emodin for 24 h	PI3K/Akt signaling pathway, TNF-α, MCP-1, IL-6	- Reduces the histopathological score of hypertrophic scars (less collagen deposition and inflammation) - Decreases recruitment and adhesion of inflammatory cells to fibroblasts - Suppresses inflammatory cytokines TNF-α and MCP-1 - Inhibits activation (phosphorylation) of PI3K and Akt, reducing inflammation and fibrosis - Exhibits low cytotoxicity and safety in fibroblast cultures	Liu (2015)
Madecassoside	Natural compound (triterpenoid saponin)	Keloid Fibroblasts, SVK-14 keratinocytes	*In Vitro*	48 h (for proliferation); 24 h (for migration)	p38 MAPK, PI3K, NF-κB, ERK	- Inhibits KF migration in scratch-wound and transwell assays - Induces KF apoptosis via mitochondrial pathway - Suppresses inflammatory signaling (e.g., TNF-α production) - Inhibits keratinocyte proliferation	Song et al. (2012)
EGCG	Green tea polyphenol	Normal fibroblasts (NFs) and Keloid fibroblasts	*In vitro* *In vivo*	*In vitro*: 25–100 μM, 1.5 h to 7 days *In vivo*: 1.25 mg/nodule, injected 7 days post-implantation, analyzed at day 17	PI3K/AKT, MEK/ERK, STAT3 signaling pathways	- Inhibits the proliferation and migration of KFs more effectively than NFs. - Induces reversible cell-cycle arrest (not apoptosis). - Reduces collagen production significantly. - Suppresses phosphorylation of AKT, ERK1/2, and STAT3. - Strongly inhibits STAT3 phosphorylation, critical for collagen production, proliferation, and migration. - Reduces keloid nodule size and collagen accumulation in vivo. - Decreases VEGF expression, suppressing angiogenesis in keloid tissue	Park et al. (2008)
Green Tea Extract (GTE)	Natural polyphenol extract	Keloid fibroblasts, HMC-1 mast cells	*In vitro*	20–80 μg/mL, 1 h pretreatment, 24 h culture	PI-3K/Akt/mTOR signaling	- Inhibits type I collagen expression - Reduces phosphorylation of Akt, 4E-BP1, and p70S6K - No cytotoxicity observed	Zhang et al. (2007)
EGCG	Natural catechin compound	Keloid fibroblasts, HMC-1 mast cells	*In vitro*	25–100 μM, 1 h pretreatment, 24 h culture	PI-3K/Akt/mTOR signaling	- Suppresses collagen synthesis in fibroblasts - Inhibits Akt/mTOR downstream effectors - Maintains high cell viability	Zhang et al. (2007)
Resveratrol (Res)	Non-flavonoid polyphenolic compound (phytoalexin)	Pathological scar fibroblasts	*In vitro*	Different concentrations, exact doses, and times are not specified clearly in the provided summary	mTOR/70S6K signaling pathway	- Reduced proliferation of pathological scar fibroblasts - Decreased expression of mTOR and 70S6K at mRNA and protein levels - Negative dose-dependent correlation with expression levels of mTOR/70S6K - Possible mechanism via suppression of mTOR/70S6K signaling pathway	Tang et al. (2017a)
Glycyrrhizin	HMGB1 Inhibitor (Natural Compound)	Keloid Fibroblasts, Human Dermal Fibroblasts (HDFs)	*In Vitro* (2D culture and 3D spheroids)	100, 200, 500 µM/48 h	HMGB1, Akt, TGF-β, ERK1/2, Smad2/3, Beclin 1, LC3	- Decreased HMGB1 expression - Reduced autophagy markers (Beclin 1, LC3) - Increased apoptosis (Annexin V, TUNEL) - Suppressed Akt, TGF-β, Smad2/3, ERK1/2 signaling- Reduced ECM deposition (collagen I/III, fibronectin, elastin)	Jeon et al. (2019)

### 5.1 Synthetic drugs

Synthetic drugs targeting the PI3K/AKT/mTOR pathway have essential roles in managing keloid formation by interfering with the intracellular signaling mechanisms that modulate cell growth, proliferation, and survival, all of which are hyperactive in keloid fibroblasts (Chen et al., 2022a). The PI3K/AKT/mTOR pathway is upregulated in keloid tissue, contributing to the excessive collagen production, abnormal fibroblast activity, and resistance to apoptosis that define keloid pathology (Kim and Kim, 2024). Synthetic drugs at this pathway work by inhibiting key enzymes, PI3K, AKT, or mTOR, thereby suppressing fibroblast proliferation, reducing ECM accumulation, and limiting scar overgrowth. These inhibitors, often used in cancer therapies, are being investigated for keloid treatment due to their potential to normalize fibroblast behavior, promote controlled healing, and reduce fibrosis and recurrence after surgical excision or injury (Tripathi et al., 2020).

#### 5.1.1 Lapatinib

Lapatinib is a dual tyrosine kinase inhibitor that targets the epidermal growth factor receptors ErbB1 (EGFR) and ErbB2 (HER2) (Patnaik et al., 2022). Lapatinib mitigates keloid fibrosis by blocking ErbB1/ErbB2 phosphorylation and subsequent PI3K/AKT activation. This inhibition reduces fibroblast proliferation, migration, and ECM synthesis, lowering levels of α-SMA, collagen I, and fibronectin. By suppressing AKT signaling, lapatinib effectively attenuates fibroblast activation and collagen deposition, alleviating keloid-associated fibrosis (Wang et al., 2025).

#### 5.1.2 Artesunate

Artesunate (ART), a well-established antimalarial drug with a favorable safety profile, has shown potent anti-fibrotic properties in treating skin hypertrophic scar (Ruwizhi et al., 2022). ART reduces hypertrophic scar formation by modulating the immune microenvironment, normalizing collagen structure, and inhibiting fibroblast activation and EMT. Its antifibrotic action arises from dual suppression of the PI3K/AKT/mTOR and TGF-β/Smad pathways, where PI3K/AKT/mTOR inhibition partly downregulates TGF-β/Smad signaling, collectively limiting ECM overproduction and scar protrusion (Shang et al., 2025).

#### 5.1.3 Remdesivir

Remdesivir (RD) is a nucleotide analog originally developed as an antiviral drug that inhibits viral RNA-dependent RNA polymerase. It is widely recognized for treating RNA virus infections like Ebola and COVID-19. Beyond its antiviral properties, recent research has revealed its potential antifibrotic effects (Li X. et al., 2021). RD effectively alleviates fibrotic responses in skin fibrosis by targeting key cellular pathways involved in fibroblast activation and ECM deposition. Specifically, RD suppresses the TGF-β1/Smad3 signaling pathway, which promotes fibroblast proliferation, migration, and transdifferentiation into myofibroblasts. Concurrently, RD inhibits the PI3K/Akt/mTOR pathway, a critical regulator of autophagy, thereby restoring autophagic flux and reducing pathological ECM accumulation (Zhang J. et al., 2024).

#### 5.1.4 Axitinib

Intralesional axitinib, a VEGFR inhibitor, effectively reduces hypertrophic scar thickness, vascularity, and collagen deposition in rabbit models without adverse effects. It exerts its antifibrotic action by suppressing angiogenesis, evidenced by reduced CD31 expression, and by inhibiting the PI3K/AKT/mTOR pathway through decreased AKT, p70S6K, and p-p70S6K levels (Liu et al., 2023).

#### 5.1.5 Sunitinib

Sunitinib is an oral multi-targeted tyrosine kinase inhibitor known for its effectiveness in treating certain cancers, such as renal cell carcinoma and gastrointestinal stromal tumors. It exerts its therapeutic action by inhibiting multiple receptor tyrosine kinases, including VEGFR, PDGFR, c-KIT, and FLT3 (Bakri et al., 2024). Sunitinib effectively suppresses keloid progression by inhibiting the overactive PI3K/Akt/mTOR pathway, leading to cell cycle arrest, apoptosis induction, and reduced fibroblast invasion. This results in markedly decreased collagen I and III synthesis. *In vitro* and *in vivo* studies, including human keloid xenografts, showed complete keloid regression with sunitinib, exceeding the antifibrotic efficacy of triamcinolone acetonide (Chen et al., 2022a).

#### 5.1.6 CUDC-907

CUDC-907 is a novel, small-molecule dual inhibitor that targets the PI3K/Akt/mTOR signaling pathway and histone deacetylases (HDACs), specifically HDAC2 (Al-Mansour et al., 2023). CUDC-907 counteracts the tumor-like behavior of keloid fibroblasts by inducing G2/M arrest (via p21 upregulation and cyclin B suppression), inhibiting proliferation, migration, invasion, and collagen I/III synthesis. It downregulates TGF-β1–Smad2/3 and Erk signaling while enhancing histone H3 acetylation. In *ex vivo* and *in vivo* models, CUDC-907 markedly reduces ECM deposition and angiogenesis in keloid tissue without altering overall tissue volume (Tu et al., 2019).

#### 5.1.7 OSI-027

OSI-027 is a second-generation mTOR kinase inhibitor that targets mTORC1 and mTORC2 complexes, offering a more comprehensive inhibition of mTOR signaling than traditional mTOR inhibitors like rapamycin (Mehta et al., 2024). Unlike rapamycin, which indirectly and incompletely inhibits mTORC1 and has no effect on mTORC2, OSI-027 directly disrupts the assembly of both mTORC1 (mTOR–Raptor) and mTORC2 (mTOR–Rictor–mLST8), thereby blocking downstream phosphorylation of key substrates such as S6K1, 4EBP1 (mTORC1 targets), and AKT (Ser-473, an mTORC2 target). This dual inhibition significantly reduces the proliferation and migration of keloid-derived keratinocytes without inducing cytotoxicity (Chen et al., 2019).

#### 5.1.8 Linagliptin

Linagliptin is a particular dipeptidyl peptidase 4 (DPP4) inhibitor, a membrane-bound enzyme that regulates cell signaling and ECM dynamics (Li et al., 2022). Linagliptin attenuates hypertrophic scar fibrosis, especially under high-glucose conditions, by inhibiting the IGF/Akt/mTOR pathway. It suppresses IGF, Akt, and mTOR phosphorylation, reducing fibroblast-to-myofibroblast transdifferentiation, proliferation, and migration. Consequently, collagen I/III and α-SMA expression decline, highlighting linagliptin’s antifibrotic potential in glucose-induced scar formation (Li et al., 2019).

#### 5.1.9 FTY720

FTY720, or fingolimod, is a pleiotropic immunomodulatory compound derived from the fungus Isaria sinclairii and approved for treating multiple sclerosis. It exerts diverse biological effects, including anti-inflammatory, anticancer, and antifibrotic actions, through modulation of sphingosine-1-phosphate receptors (S1PRs), particularly S1PR5 (Pournajaf et al., 2022). FTY720 exerts potent antifibrotic effects in hypertrophic scars and keloids by inducing fibroblast cell cycle arrest, promoting apoptosis, and reducing migration, contraction, and collagen I/III deposition. Acting through S1PR5, it inhibits the PI3K/Akt/mTOR/p70S6K pathway, independent of Smad-mediated TGF-β signaling, thereby suppressing fibroblast activation and promoting scar resolution *in vitro* and *in vivo* (Shi et al., 2017).

#### 5.1.10 17-AAG

17-AAG (17-allylaminodemethoxygeldanamycin) is a small-molecule inhibitor that targets heat shock protein 90 (HSP90), a molecular chaperone involved in the stabilization and function of various client proteins essential for cell growth, survival, and migration (Talaei et al., 2019). In keloid fibroblasts with elevated HSP90 levels, 17-AAG disrupts HSP90 activity, causing degradation of client proteins such as Akt. This suppression of Akt signaling enhances apoptosis and markedly reduces fibroblast proliferation and migration, highlighting 17-AAG as a promising therapeutic agent for correcting the pathological hyperactivity of keloid fibroblasts (Yun et al., 2015).

#### 5.1.11 KU-0063794 and KU-0068650

KU-0063794 and KU-0068650, dual mTORC1/2 inhibitors, suppress the PI3K/Akt/mTOR pathway by blocking Akt Ser473 phosphorylation. In keloid models, they markedly reduce fibroblast viability, proliferation, migration, invasion, and ECM protein expression (collagen, fibronectin, α-SMA), while promoting apoptosis and tissue shrinkage at low doses (2.5–10 µM). Additionally, they exert potent anti-angiogenic effects by depleting CD31^+^/CD34^+^ endothelial cells, highlighting their strong antifibrotic potential (Syed et al., 2013).

#### 5.1.12 P529

P529, a dual mTORC1/2 inhibitor, effectively blocks the overactive PI3K/Akt/mTOR pathway in keloids. By disrupting both mTOR complexes, it suppresses phosphorylation of Akt (Ser473), mTOR, and downstream effectors pS6 and 4E-BP1. Unlike rapamycin, which targets only mTORC1 and may cause Akt reactivation, P529 fully inhibits the pathway, offering a more comprehensive antifibrotic effect (Weinberg, 2016). This broad inhibition reduces fibroblast proliferation, migration, invasion, ECM production (collagen I, fibronectin, and α-SMA), and angiogenesis, while inducing apoptosis selectively in keloid fibroblasts (Syed et al., 2012).

#### 5.1.13 LSKL

LSKL is a peptide that is a selective antagonist of thrombospondin-1 (TSP-1), a key activator of latent TGF-β, which is heavily implicated in fibrotic processes like hypertrophic scars and keloids (Xie et al., 2010). LSKL inhibits TSP-1–mediated TGF-β activation, thereby reducing fibroblast activation and ECM overproduction. It markedly lowers collagen I and α-SMA expression in hypertrophic scar fibroblasts *in vitro* and *in vivo*. Its antifibrotic action occurs through suppression of the PI3K/AKT/mTOR pathway, independent of SMAD or MAPK signaling (Xu X. et al., 2020).

### 5.2 Hydrogels

Hydrogels represent a promising therapeutic platform for keloid management, owing to their excellent biocompatibility, moisture retention, and capacity for sustained, localized drug delivery (Xu et al., 2025). Three-dimensional porous polymer scaffolds can be designed to deliver anti-inflammatory or antifibrotic agents, stem cells, or nucleic acids to counter keloid hallmarks, fibroblast overgrowth, collagen excess, and chronic inflammation. Smart, stimuli-responsive hydrogels further enhance therapy by enabling controlled drug release and reducing skin tension, helping prevent keloid recurrence (Zhong et al., 2024). The LA-peptide hydrogel is a self-assembling, injectable, and biocompatible system that promotes scarless healing by modulating the wound microenvironment. It forms a nanonet structure allowing sustained peptide release and adsorbs excess TGF-β, thereby preventing M2 macrophage polarization and fibroblast-to-myofibroblast activation. By downregulating TGF-β/Smad2/3 and PI3K/Akt pathways, it disrupts the profibrotic macrophage–fibroblast feedback loop and limits collagen deposition (Li Z. et al., 2025). The RGD/CS/β-GP bio-hydrogel is a thermosensitive, injectable system combining chitosan and β-glycerophosphate to form a porous, biocompatible matrix at body temperature. Incorporation of RGD peptides enhances integrin-mediated cell signaling and controlled peptide release. In keloids and postoperative adhesion prevention, it downregulates SMAD, PI3K, and P38 MAPK pathways, thereby reducing fibroblast overactivity, inflammation, and fibrosis, ultimately preventing abnormal scar formation (Liang et al., 2025).

### 5.3 Liposome

Paclitaxel–cholesterol liposomes (PTXLs) enhance paclitaxel’s stability, bioavailability, and sustained release for targeted antifibrotic therapy. In keloid fibroblasts, PTXLs outperform free PTX by inhibiting proliferation, inducing apoptosis, and reducing invasiveness. They suppress TNF-α, IL-6, and TGF-β via PI3K/AKT/GSK3β inhibition, leading to decreased α-SMA and collagen I expression and effectively mitigating keloid fibrosis (Wang et al., 2019).

### 5.4 Stem cells

Adipose-derived stem cell (ADSC) exosomes exert antifibrotic effects in keloids by restoring mitochondrial autophagy. They suppress the overactive PI3K/AKT/mTOR pathway, promoting the clearance of damaged mitochondria, reducing oxidative stress, and improving mitochondrial function. This leads to decreased inflammation, reduced collagen deposition, and enhanced scar remodeling, underscoring their therapeutic promise for keloid treatment (Liu et al., 2024). ADSCs inhibit keloid fibroblast proliferation and induce apoptosis through suppression of ITGA2, a collagen-binding receptor central to ECM remodeling and fibrosis. Overexpression of ITGA2 negates these antifibrotic effects, confirming its pivotal role. ADSCs also modulate the PI3K/Akt pathway, regulating fibroblast survival, proliferation, and adhesion, thereby contributing to their therapeutic impact in keloid pathology (Chen et al., 2022b).

### 5.5 Microneedle

Microneedles (MNs) are tiny, minimally invasive devices (25–1000 µm) designed to penetrate the skin painlessly and deliver therapeutic agents directly to targeted sites. In keloid treatment, MNs enable precise delivery of anti-scar compounds through dense fibrotic tissue, enhancing drug absorption and therapeutic efficacy while minimizing discomfort (Mbituyimana et al., 2023). Glabridin-loaded dissolving microneedles (Gla-MNs) enhance keloid therapy by delivering glabridin directly into the dermis through a dissolvable hyaluronic acid–polyvinyl alcohol matrix. This system improves drug absorption, suppresses fibroblast proliferation, and reduces collagen overproduction. Mechanistically, Gla-MNs inhibit the PI3K/Akt and TGF-β1/α-SMA pathways, decreasing phosphorylation of PI3K/Akt, promoting fibroblast apoptosis, and limiting ECM accumulation (Guo et al., 2024).

### 5.6 Nanoparticles

Nanoparticles (1–100 nm) serve as advanced drug delivery systems in keloid therapy, offering controlled, targeted, and sustained release of therapeutic agents to affected skin layers. They enhance drug stability, penetration, and cellular uptake while reducing systemic toxicity. When integrated with microneedles or photodynamic therapy, functionalized nanoparticles enable precise targeting, disrupt collagen overproduction, inhibit fibroblast proliferation, and modulate inflammation, improving overall antifibrotic efficacy (Chen Z. et al., 2024). Porous Se@SiO_2_ nanoparticles feature a selenium core within a silica shell, enabling controlled antioxidant release and improved stability. By maintaining optimal ROS levels, they reduce oxidative stress, prevent fibroblast apoptosis, and limit excessive ECM deposition. Through activation of the PI3K/Akt pathway, Se@SiO_2_ NPs promote fibroblast survival, inhibit myofibroblast differentiation, and enhance wound healing while minimizing fibrosis (Yang et al., 2022).

### 5.7 Botulinum toxin type A

Botulinum toxin type A (BTXA) is a neurotoxin derived from *Clostridium botulinum*, widely used in clinical treatments for conditions like hyperhidrosis, muscle spasticity, and increasingly, hypertrophic scars and keloids (Austin et al., 2018). BTXA inhibits TGF-β1–induced fibroblast-to-myofibroblast differentiation by downregulating collagen I/III and α-SMA, suppressing proliferation, and promoting apoptosis. Mechanistically, it prevents PTEN methylation, restores PTEN expression, and reduces DNA methyltransferase activity. This reactivation of PTEN downregulates PI3K/Akt phosphorylation, effectively restraining fibroblast activation and fibrosis progression in keloids (Zhang X. et al., 2019).

### 5.8 Photoelectric therapy

Photoelectric (laser-based) therapy treats pathological scars by selectively damaging microvessels, reducing collagen synthesis, inducing fibroblast apoptosis, and remodeling collagen fibers through thermal and photomechanical effects (Chen SX. et al., 2022). Mechanistically, laser exposure upregulates miR-206 and downregulates mTOR signaling, suppressing fibroblast proliferation and enhancing apoptosis. This miR-206/mTOR axis modulation underlies the therapy’s antifibrotic efficacy in hypertrophic scars and keloids (Zhang et al., 2020).

### 5.9 Electron beam irradiation

Electron beam (EB) irradiation reduces keloid recurrence by inducing apoptosis in hyperproliferative fibroblasts (Yan et al., 2020). It suppresses miR-21-5p, leading to PTEN upregulation and inhibition of the AKT pathway. This decreases p-AKT and LC3B-II levels, suppressing autophagy and fibroblast migration. Through the miR-21-5p/PTEN/AKT axis, EB irradiation limits fibroblast survival, invasion, and keloid recurrence (Yan et al., 2020).

### 5.10 Natural products

Natural products offer multifaceted therapeutic potential in keloid management by modulating fibroblast proliferation, collagen synthesis, inflammation, and key signaling pathways such as TGF-β/Smad, IL-6/STAT3, and PI3K/Akt/mTOR. Their antioxidant, anti-inflammatory, and antifibrotic properties, combined with low toxicity and multitarget activity, make them effective candidates for treating the complex pathophysiology of keloids (Song et al., 2024).

#### 5.10.1 WuFuYin

WuFuYin (WFY), a traditional Chinese herbal formula composed of Panax ginseng, Rehmanniae Radix Praeparata, Angelica sinensis, Atractylodes macrocephala, and licorice, exhibits immunoregulatory and anti-inflammatory effects relevant to scar pathology (Li et al., 2025c). In hypertrophic scars and keloid-like fibrosis, WFY’s active compounds, such as (2R)-7-hydroxy-2-(4-hydroxyphenyl)chroman-4-one and prenylated eriodictyols, target CDK2, GSK3B, and PIK3CG within the PI3K/Akt pathway. By modulating this signaling cascade, WFY inhibits fibroblast proliferation and inflammation, reducing excessive ECM deposition and fibrosis (Zhang S-Y. et al., 2024).

#### 5.10.2 Shikonin

Shikonin (SHI), a naphthoquinone compound with anti-inflammatory and antitumor properties, promotes scar repair in hypertrophic scarring by inducing autophagy in fibroblasts (Song et al., 2023). It activates the AMPK/mTOR pathway, enhancing AMPK phosphorylation and suppressing mTOR, thereby increasing LC3-II and Beclin1 while reducing P62. This autophagic activation inhibits fibroblast proliferation and ECM deposition, positioning SHI as a promising antifibrotic agent for hypertrophic scars (Zhang Q. et al., 2023).

#### 5.10.3 Silibinin

Silibinin, a flavonoid from milk thistle with potent antifibrotic activity, inhibits keloid progression by suppressing the mTOR signaling pathway. It reduces phosphorylation of mTOR and downstream effectors (p70S6K, S6, 4E-BP1), leading to dose-dependent decreases in collagen I and III expression in both normal and keloid fibroblasts. By limiting ECM production and fibroblast overactivity, silibinin shows strong therapeutic potential for treating pathological scarring (Choi et al., 2023).

#### 5.10.4 Melatonin

Melatonin, a pineal hormone with antioxidant and anti-fibrotic properties, attenuates hypertrophic scar formation by suppressing fibroblast proliferation, migration, and contractility. It downregulates collagen I, collagen III, and α-SMA while enhancing autophagic flux via MT2 receptor activation. By inhibiting the PI3K/Akt/mTOR pathway, an autophagy suppressor, melatonin promotes degradation of fibrotic proteins, thereby reducing fibroblast activity and limiting scar progression (Dong et al., 2024). Similarly, melatonin regulates keloid fibroblast activity mainly through MT2 receptor–mediated inhibition of the cAMP/PKA/Erk and Smad pathways, with minimal impact on Akt/mTOR signaling. By reducing cAMP and PKA activity, it suppresses BRAF and Erk phosphorylation, leading to decreased fibroblast proliferation, migration, invasion, and collagen synthesis. Concurrently, melatonin blocks TGF-β1–induced Smad2/3 phosphorylation, preventing myofibroblast differentiation and ECM accumulation, thereby exerting potent antifibrotic effects independent of the PI3K/Akt/mTOR pathway (Huang S. et al., 2023).

#### 5.10.5 Glabridin

Glabridin (Gla), a flavonoid from licorice, exhibits potent antifibrotic effects against keloid formation by inhibiting fibroblast proliferation and inducing apoptosis (Guo et al., 2024). It suppresses the PI3K/Akt pathway to reduce cell survival and blocks TGF-β1/Smad signaling, decreasing α-SMA, COL1A1, and COL3A1 expression. The reversal of these effects by Akt and TGF-β agonists confirms that Gla acts through coordinated inhibition of both PI3K/Akt and TGF-β1/Smad pathways to limit fibrosis (Zhang et al., 2022).

#### 5.10.6 Glycyrrhizin

Glycyrrhizin, a licorice-derived compound, inhibits HMGB1, a key mediator of inflammation, fibrosis, and autophagy in keloids. Blocking HMGB1 activity reduces autophagy (lower Beclin 1 and LC3 expression) and promotes apoptosis in keloid fibroblasts and spheroids. This effect is linked to suppression of the HMGB1-Akt signaling axis, leading to decreased fibroblast survival, reduced ECM deposition, and attenuation of fibrotic progression (Jeon et al., 2019).

#### 5.10.7 Panax Notoginseng Saponins

Panax Notoginseng Saponins (PNS) attenuate hypertrophic scar formation by downregulating TRPM7, a key regulator of fibroblast proliferation and ECM synthesis. This inhibition suppresses the PI3K/Akt pathway, leading to decreased fibroblast growth, enhanced apoptosis, and reduced collagen production. Through these mechanisms, PNS exhibit strong anti-fibrotic potential, supporting their therapeutic use in managing hypertrophic scars (Zhi et al., 2021).

#### 5.10.8 Wubeizi ointment

Wubeizi ointment, containing Salvia miltiorrhiza, Clematis, and Galla Chinensis, exerts strong antifibrotic effects against keloid formation by inhibiting the Akt/mTOR pathway. It downregulates phosphorylated Akt and mTOR while upregulating PTEN, leading to reduced fibroblast proliferation and enhanced apoptosis in a dose-dependent manner. The reversal of these effects by IGF-1 confirms that Wubeizi’s therapeutic action is mediated through suppression of the Akt/mTOR signaling cascade (Tang et al., 2020a).

#### 5.10.9 Tetramethylpyrazine

Tetramethylpyrazine (TMP), an active compound from Chuanxiong Rhizoma (Yang et al., 2021), exhibits potent antifibrotic effects in hypertrophic scars by inhibiting fibroblast proliferation and enhancing apoptosis. It reduces collagen I, collagen III, and α-SMA expression, limiting ECM accumulation. TMP induces G0/G1 cell cycle arrest and modulates mitochondrial apoptosis by increasing Bax and cleaved Caspase-3 while decreasing Bcl-2. Mechanistically, it suppresses the PI3K/Akt pathway through dose-dependent inhibition of Akt phosphorylation at Ser473, thereby attenuating fibrotic activity (Wu et al., 2020).

#### 5.10.10 Galla Chinensis ointment

Galla Chinensis ointment is a traditional Chinese medicine formulation composed of ingredients such as Galla Chinensis, Salvia miltiorrhiza, medlar, clematis, and black vinegar, prepared using modern pharmaceutical processing techniques (Meetam et al., 2024). Galla Chinensis ointment shows strong clinical efficacy (96.6%) against keloids by modulating the PI3K/Akt/mTOR pathway. It downregulates miR-21, restoring PTEN expression and consequently reducing Akt and mTOR phosphorylation. This suppresses fibroblast proliferation and limits keloid progression, highlighting its potent antifibrotic mechanism via miR-21/PTEN/PI3K/Akt regulation (Tang et al., 2020b).

#### 5.10.11 Pentoxifylline (PTX)

Pentoxifylline (PTX) is a methylxanthine derivative with antioxidant and anti-inflammatory properties that has shown therapeutic potential in treating HTS (Ahmadi and Khalili, 2016). PTX suppresses hypertrophic scar formation by inhibiting fibroblast proliferation and ECM production via the PI3K/Akt/FoxO/p27^Kip1 pathway. It decreases Akt phosphorylation, reactivating FoxO1 and increasing p27^Kip1 expression, which induces G1 cell cycle arrest and reduces collagen I, collagen III, and α-SMA levels. *In vitro* and *in vivo*, PTX markedly lowers collagen deposition and dermal thickening, highlighting its therapeutic potential in regulating fibroblast activity through Akt/FoxO/p27^Kip1 signaling (Yang et al., 2019).

#### 5.10.12 Quercetin

Quercetin, a natural flavonoid found in a variety of fruits and vegetables, is well known for its antioxidant and anti-inflammatory properties (Azeem et al., 2023). Quercetin enhances the radiosensitivity of keloid fibroblasts by promoting apoptosis through PI3K/Akt-dependent inhibition of HIF-1α. It decreases phosphorylated Akt and HIF-1α levels, weakening fibroblast resistance to radiation-induced stress. The partial reversal of these effects by IGF-1 confirms Akt involvement. By suppressing the PI3K/Akt/HIF-1α axis, quercetin sensitizes keloid fibroblasts to ionizing radiation, improving therapeutic efficacy (Si L-B. et al., 2018).

#### 5.10.13 Catechols

Catechols like caffeic acid (CA) and 3,4-dihydroxyphenylethanol (DOPE), key aglycon components of acteoside, regulate ECM remodeling by promoting proMMP-2 activation and upregulating MT1-MMP in dermal fibroblasts. Unlike modified catechols, CA and DOPE specifically act through the PI3K pathway, as PI3K inhibition suppresses these effects. This highlights their crucial role in PI3K-dependent modulation of fibroblast-driven ECM degradation during wound repair (Si N. et al., 2018).

#### 5.10.14 Gallic acid

Gallic acid (GA), a plant-derived polyphenol with strong antioxidant and anti-inflammatory properties, inhibits keloid fibroblast proliferation, migration, and invasion by inducing G1 cell cycle arrest and apoptosis (Merecz-Sadowska et al., 2021). It suppresses the AKT/ERK pathway, reducing cell survival and growth, and modulates ECM remodeling by downregulating MMP-1/MMP-3 and upregulating TIMP-1. Additionally, GA lowers VEGF and VEGFR expression, diminishing angiogenesis and reinforcing its antifibrotic and anti-keloid potential (Wang et al., 2018).

#### 5.10.15 Naringin

Naringin, a citrus-derived flavonoid, exhibits potent antifibrotic effects by inhibiting hypertrophic scar fibroblast proliferation, migration, and survival. It induces cell cycle arrest and apoptosis in a dose-dependent manner. Mechanistically, naringin selectively suppresses Akt kinase activity, reducing its phosphorylation at Ser473 and Thr308 and downregulating downstream signaling (Song et al., 2018).

#### 5.10.16 Emodin

Emodin, a rhubarb-derived bioactive compound, mitigates hypertrophic scarring by suppressing mechanical stress-induced inflammation and fibrosis. It reduces collagen accumulation, inflammatory cell infiltration, and fibroblast–immune cell interactions, leading to decreased TNF-α and MCP-1 production. These antifibrotic and anti-inflammatory effects are primarily mediated through inhibition of the PI3K/Akt pathway, a central regulator of scar-associated inflammation and tissue remodeling (Liu, 2015).

#### 5.10.17 Madecassoside

Madecassoside, a triterpenoid saponin from *Centella asiatica*, exerts potent anti-keloid effects by inhibiting fibroblast proliferation and migration. It suppresses the p38 MAPK and PI3K signaling pathways, key regulators of inflammation, cell motility, and survival. Through this dual inhibition, madecassoside disrupts the aberrant wound healing processes that drive keloid formation, underscoring its promise as a targeted antifibrotic therapy (Song et al., 2012).

#### 5.10.18 Epigallocatechin-3-gallate

Epigallocatechin-3-gallate (EGCG), the main polyphenol in green tea, exhibits strong antifibrotic effects in keloids by inhibiting fibroblast proliferation, migration, and collagen synthesis. It suppresses the PI3K/Akt and STAT3 pathways, reducing phosphorylation of PI3K/Akt and downregulating cyclin D1 and c-Myc to halt cell cycle progression. EGCG’s potent inhibition of STAT3 activation, confirmed by inhibitor and siRNA studies, further decreases collagen expression, highlighting its therapeutic potential for controlling keloid development (Park et al., 2008). Green tea extract (GTE) and its major polyphenol, EGCG, inhibit mast cell–induced collagen I production in keloid fibroblasts by suppressing the PI3K/Akt/mTOR pathway. In mast cell–fibroblast co-culture models, GTE and EGCG reduce phosphorylation of Akt, 4E-BP1, and p70S6K in a dose-dependent manner, thereby limiting collagen synthesis without cytotoxic effects (Zhang et al., 2007).

#### 5.10.19 Resveratrol

Resveratrol, a natural polyphenol with anti-inflammatory and antiproliferative properties, suppresses pathological scar fibroblast growth by inhibiting the Akt/mTOR signaling pathway. It downregulates Akt and mTOR expression in a dose-dependent manner, while exerting minimal effect on PI3K. This selective inhibition underscores resveratrol’s potential as a therapeutic agent for controlling fibroblast hyperproliferation and pathological scar formation (Tang Z. et al., 2017). Resveratrol (Res) exhibits diverse biological activities, including anti-inflammatory, antioxidant, and mitochondrial-protective effects. In pathological scar fibroblasts, it inhibits proliferation by downregulating mTOR and its downstream effector 70S6K, both of which are upregulated compared to normal fibroblasts. Res treatment decreases mTOR and 70S6K expression in a dose-dependent manner, highlighting its potential to suppress pathological scar formation through targeted inhibition of the mTOR signaling pathway (Tang Z-M. et al., 2017).

## 6 Conclusion

In summary, the PI3K/AKT/mTOR signaling axis emerges as a central driver of the aberrant fibroproliferative response in both keloids and hypertrophic scars, orchestrating key pathogenic processes, fibroblast hyperplasia, resistance to apoptosis, excessive ECM deposition, and metabolic reprogramming. Molecular studies demonstrate that overactivation of PI3K/AKT/mTOR promotes the synthesis of collagen types I and III, myofibroblast differentiation (α-SMA upregulation), and inhibition of autophagy (via ULK1 suppression), thereby perpetuating chronic inflammation and scar expansion. Upstream modulators such as HMGB1, DJ–1–mediated PTEN nitrosylation, NEDD4-driven PTEN ubiquitination, RAGE/IGF-1/CD26 signaling, and non-coding RNAs (e.g., miR-21, miR-203a-3p, circCOL5A1) converge on this pathway, amplifying fibrogenic cues and metabolic shifts (Warburg effect) that mirror quasi-neoplastic behavior. Therapeutically, targeted inhibition of PI3K, AKT, or mTOR demonstrates robust anti-scar efficacy across multiple modalities. Small-molecule inhibitors (OSI-027, KU-0063794/650, P529) and dual-HDAC/PI3K inhibitors (CUDC-907) arrest cell cycle progression, induce fibroblast apoptosis, and collapse keloid explants. Repositioned drugs (sunitinib, lapatinib, remdesivir, artesunate) similarly reduce scar thickness and collagen deposition in preclinical models. At the same time, natural compounds (silibinin, glabridin, EGCG, melatonin) effectively downregulate mTOR signaling and restore autophagic flux. Emerging delivery systems, such as hydrogels, microneedles, liposomes, and exosome-based approaches, enhance local bioavailability, yielding sustained pathway suppression and improved remodeling. Collectively, these findings validate PI3K/AKT/mTOR as both a mechanistic nexus and a versatile target for scar intervention. Future work should prioritize translational studies and controlled clinical trials to optimize dosing, delivery, and combination regimens, transforming existing management paradigms toward scar-free wound healing. Despite compelling preclinical evidence, several limitations temper the translation of PI3K/AKT/mTOR-targeted therapies to clinical practice. First, many studies rely on single-cell types or animal models that do not fully recapitulate human keloid heterogeneity, mechanical stresses, or immune microenvironment. Second, systemic toxicity and off-target effects of potent kinase inhibitors (e.g., P529, OSI-027) remain concerns, necessitating optimized dosing and localized delivery strategies. Third, the redundancy and feedback loops within PI3K/AKT/mTOR and parallel pathways (e.g., TGF-β/Smad, MAPK) may limit monotherapy efficacy and promote resistance. Future research should focus on developing patient-derived 3D organoids and *ex vivo* explant platforms to better model keloid biology; engineering targeted delivery vehicles (e.g., ligand-functionalized nanoparticles, dissolving microneedles) to enhance local bioavailability while minimizing systemic exposure; exploring combination regimens that co-target complementary fibrotic pathways (e.g., PI3K/mTOR plus TGF-β or JAK/STAT inhibitors); and identifying predictive biomarkers (e.g., circulating miRNAs, phospho-AKT levels) to stratify patients and monitor therapeutic response. The field can advance from mechanistic insights to safe, effective, personalized scar-modulating therapies by addressing these gaps.

## Glossary

17-AAG: 17-allylamino-17-demethoxygeldanamycin (HSP90 inhibitor)
4EBP1: eIF4E-binding protein 1
ADSC: Adipose-derived stem cell
ADSCs: Adipose-derived stem cells
AKT: Protein kinase B (Akt)
AKT1: AKT serine/threonine kinase 1
ALDH1: Aldehyde dehydrogenase 1
AMPK: AMP-activated protein kinase
ANGPT2: Angiopoietin-2
ART: Artesunate
ATP: Adenosine triphosphate
AURKA: Aurora kinase A
Akt: Protein kinase B
BRAF: B-Raf proto-oncogene, serine/threonine kinase
BTXA: Botulinum toxin type A
CCN2: Cellular communication network factor 2 (CTGF)
CD26: Cluster of differentiation 26 (DPP4)
CD31: Cluster of differentiation 31
CD34: Cluster of differentiation 34
CD44: Cluster of differentiation 44
CD8: Cluster of differentiation 8
CDK2: Cyclin-dependent kinase 2
COL1: Collagen type I
COL1A1: Collagen type I alpha 1 chain
COL3: Collagen type III
COL3A1: Collagen type III alpha 1 chain
CTGF: Connective tissue growth factor
CUDC-907: Dual PI3K/Akt/mTOR and HDAC inhibitor
CXCL2: C-X-C motif chemokine ligand 2
CXCR7: C-X-C chemokine receptor 7
CYLD: Cylindromatosis (deubiquitinase)
DJ-1: Parkinsonism associated deglycase (DJ-1/PARK7)
DNA: Deoxyribonucleic acid
DOPE: 3,4-dihydroxyphenylethanol
DPP4: Dipeptidyl peptidase-4
EB: Electron beam
ECAR: Extracellular acidification rate
ECM: Extracellular matrix
EGCG: Epigallocatechin-3-gallate
EGFR: Epidermal growth factor receptor
ELFs: Extra-lesional fibroblasts
EMT: Epithelial–mesenchymal transition
ERK: Extracellular signal-regulated kinase
ERK1: Extracellular signal-regulated kinase 1
ESI-09: Epac-specific inhibitor ESI-09
Epac1: Exchange protein directly activated by cAMP 1
FAK: Focal adhesion kinase
FLT3: Fms-like tyrosine kinase 3
FN1: Fibronectin 1
FOXO3a: Forkhead box O3
FPASL: Fibroblast proliferation-associated lncRNA
FTY720: Fingolimod
GAB1: GRB2-associated-binding protein 1
GLUT1: Glucose transporter 1
GSK3B: Glycogen synthase kinase-3 beta
GSK3β: Glycogen synthase kinase-3 beta
GTE: Green tea extract
GW4869: Neutral sphingomyelinase inhibitor (exosome release inhibitor)
HDAC: Histone deacetylase
HDAC2: Histone deacetylase 2
HER2: Human epidermal growth factor receptor 2 (ERBB2)
HER3: Human epidermal growth factor receptor 3 (ERBB3)
HIF-1: Hypoxia-inducible factor-1
HIF-1α: Hypoxia-inducible factor-1 alpha
HK2: Hexokinase 2
HKFs: Human keloid fibroblasts
HMC-1: Human mast cell line-1
HMGB1: High-mobility group box 1
HSP90: Heat shock protein 90
HTS: Hypertrophic scars
HTSF: Hypertrophic scarring fibroblasts
HUVECs: Human umbilical vein endothelial cells
HVA: Homovanillic acid
Hsp90: Heat shock protein 90
IGF: Insulin-like growth factor
IGF-1: Insulin-like growth factor 1
IGF-1R: Insulin-like growth factor 1 receptor
IGF1: Insulin-like growth factor 1 (gene)
IL-10: Interleukin-10
IL-13: Interleukin-13
IL-18: Interleukin-18
IL-6: Interleukin-6
IL-8: Interleukin-8
ITGA2: Integrin subunit alpha 2
ITGAV: Integrin subunit alpha V
JAK: Janus kinase
JNK: c-Jun N-terminal kinase
KIT: Tyrosine-protein kinase KIT (c-Kit, CD117)
KU-0063794: ATP-competitive mTORC1/2 inhibitor
KU-0068650: ATP-competitive mTORC1/2 inhibitor
LC3-II: Microtubule-associated protein 1 light chain 3-II
LC3B-II: Microtubule-associated protein 1 light chain 3B-II
LINC00173: Long intergenic non-protein coding RNA 173
LINC01605: Long intergenic non-protein coding RNA 1605
LY294002: PI3K inhibitor
MAPK: Mitogen-activated protein kinase
MCP-1: Monocyte chemoattractant protein-1 (CCL2)
MEK: MAPK/ERK kinase
MMP-1: Matrix metalloproteinase-1
MMP-2: Matrix metalloproteinase-2
MMP-9: Matrix metalloproteinase-9
MNs: Microneedles
MOPE: 3-methoxy-4-hydroxyphenylethanol
MT1-MMP: Membrane-type 1 matrix metalloproteinase (MMP-14)
MT2: Melatonin receptor 2 (MTNR1B)
MTT: Tetrazolium (MTT) cell viability assay
NDRG2: N-Myc downstream-regulated gene 2
NEDD4: E3 ubiquitin-protein ligase NEDD4
NEDD4-1: E3 ubiquitin-protein ligase NEDD4-1
NF-κB: Nuclear factor kappa-B
NFs: Normal fibroblasts
NHDF: Normal human dermal fibroblasts
NOTCH: Notch receptor signaling
NPs: Nanoparticles
NRF2: Nuclear factor erythroid 2–related factor 2
NRG1: Neuregulin 1
OCR: Oxygen consumption rate
OSI-027: mTOR kinase inhibitor (dual mTORC1/2)
PARP: Poly(ADP-ribose) polymerase
PCK: Protein kinase C
PCR: Polymerase chain reaction
PDGFR: Platelet-derived growth factor receptor
PDK1: 3-Phosphoinositide-dependent protein kinase-1
PFKFB3: 6-phosphofructo-2-kinase/fructose-2,6-bisphosphatase 3
PHLPP2: PH domain and leucine-rich repeat protein phosphatase 2
PI3K: Phosphoinositide 3-kinase
PIK3CA: Phosphatidylinositol-4,5-bisphosphate 3-kinase catalytic subunit alpha
PIK3CG: Phosphatidylinositol-4,5-bisphosphate 3-kinase catalytic subunit gamma
PIP2: Phosphatidylinositol (4,5)-bisphosphate
PIP3: Phosphatidylinositol (3,4,5)-trisphosphate
PKA: Protein kinase A
PLK1: Polo-like kinase 1
PNS: Panax notoginseng saponins
PTEN: Phosphatase and tensin homolog
PTEN-PI3K: PTEN–PI3K axis
PTX: Paclitaxel
PTXL: Paclitaxel–cholesterol liposomes
PTXLs: Paclitaxel–cholesterol liposomes
RAGE: Receptor for advanced glycation end products
RAGE-MAPK: RAGE–MAPK signaling axis
RNA: Ribonucleic acid
Raptor: Regulatory-associated protein of mTOR
Rheb: Ras homolog enriched in brain
Rictor: Rapamycin-insensitive companion of mTOR
S1PR5: Sphingosine-1-phosphate receptor 5
S6K1: Ribosomal protein S6 kinase 1
SHI: Shikonin
SMAD: SMAD family proteins
SMAD2: SMAD family member 2
STAT: Signal transducer and activator of transcription (family)
STAT3: Signal transducer and activator of transcription 3
STING: Stimulator of interferon genes
Se@SiO2: Selenium-in-silica (porous Se@SiO2) nanoparticles
TGFBR2: Transforming growth factor beta receptor 2
TIMP-1: Tissue inhibitor of metalloproteinases-1
TM4SF1: Transmembrane 4 L six family member 1
TMP: Tetramethylpyrazine
TRPM7: Transient receptor potential melastatin 7
TSC2: Tuberous sclerosis complex 2
TSP-1: Thrombospondin-1
TSV1: Transcript splice variant 1
TSV2: Transcript splice variant 2
TUNEL: Terminal deoxynucleotidyl transferase dUTP nick end labeling
ULK1: Unc-51 like autophagy activating kinase 1
USP37: Ubiquitin-specific protease 37
UTR: Untranslated region
VEGF: Vascular endothelial growth factor
VEGFR: Vascular endothelial growth factor receptor
VEGFRs: Vascular endothelial growth factor receptors
WFY: WuFuYin (herbal formula)
c-Met: Hepatocyte growth factor receptor (c-Met)
cGAS: Cyclic GMP-AMP synthase
circRNA: Circular RNA
lncRNA: Long non-coding RNA
mLST8: mTOR associated protein, LST8 homolog
mTOR: Mechanistic target of rapamycin
mTORC1: mTOR complex 1
mTORC2: mTOR complex 2
p38: p38 mitogen-activated protein kinase
p70S6K: 70-kDa ribosomal S6 kinase
p75NTR: p75 neurotrophin receptor (NGFR)
α-SMA: Alpha-smooth muscle actin
